# Metabolic Profiling, Antiviral Activity and the Microbiome of Some Mauritian Soft Corals

**DOI:** 10.3390/md21110574

**Published:** 2023-10-31

**Authors:** Deeya Jahajeeah, Mala Ranghoo-Sanmukhiya, Georgia Schäfer

**Affiliations:** 1 Department of Agricultural & Food Science, Faculty of Agriculture, University of Mauritius, Reduit 80837, Mauritius; m.sanmukhiya@uom.ac.mu; 2 International Centre for Genetic Engineering and Biotechnology, Cape Town 7925, South Africa; georgia.schafer@icgeb.org

**Keywords:** Mauritius, soft corals, metabolic profiling, metagenomics, antiviral, SARS-CoV-2, HPV pseudovirus

## Abstract

Soft corals, recognized as sessile marine invertebrates, rely mainly on chemical, rather than physical defense, by secreting intricate secondary metabolites with plausible pharmaceutical implication. Their ecological niche encompasses a diverse community of symbiotic microorganisms which potentially contribute to the biosynthesis of these bioactive metabolites. The emergence of new viruses and heightened viral resistance underscores the urgency to explore novel pharmacological reservoirs. Thus, marine organisms, notably soft corals and their symbionts, have drawn substantial attention. In this study, the chemical composition of four Mauritian soft corals: *Sinularia polydactya*, *Cespitularia simplex*, *Lobophytum patulum*, and *Lobophytum crassum* was investigated using LC–MS techniques. Concurrently, Illumina 16S metagenomic sequencing was used to identify the associated bacterial communities in the named soft corals. The presence of unique biologically important compounds and vast microbial communities found therein was further followed up to assess their antiviral effects against SARS-CoV-2 and HPV pseudovirus infection. Strikingly, among the studied soft corals, *L. patulum* displayed an expansive repertoire of unique metabolites alongside a heightened bacterial consort. Moreover, *L. patulum* extracts exerted some promising antiviral activity against SARS-CoV-2 and HPV pseudovirus infection, and our findings suggest that *L. patulum* may have the potential to serve as a therapeutic agent in the prevention of infectious diseases, thereby warranting further investigation.

## 1. Introduction

Mauritius Island is a volcanic island, which has one of the biggest Exclusive Economic Zones at 2.3 million square kilometers, in the Indian Ocean. The coral reefs surrounding Mauritius Island still need to be fully explored, as they hold many unexplored marine compounds. Soft corals (class Octocorallia) form an integral part of the island’s reef ecosystem [1], and their importance range from providing a habitat to other marine organisms to being a bountiful source of new marine natural products, and one of the prominent bioactive secondary metabolites’ sources [2]. Soft corals can produce a broad variety of chemical compounds with unique chemical structures and bioactive characteristics which could lead to the successful development of commercial drugs. The bioactive secondary metabolites, such as terpenoids, cembranoids, and steroids, from soft corals have exhibited interesting biological properties, including cytotoxic, antifungal, antibacterial, anti-inflammatory, and antiviral activity [3,4,5,6].

Furthermore, there is evidence that soft corals are prolific producers of secondary metabolites [6,7]. Interestingly, the presence of symbiotic microorganisms in soft corals has raised many debates on the origin of the secondary metabolites of the hosts. It has been recognized that dense and diverse microbial communities harboring on and in the tissues of soft corals are the true producers of many metabolites responsible for antiviral, anticancer, and anti-inflammatory properties [8]. 

The rise of emerging viruses, such as SARS-CoV-2, draws the attention of scientists throughout the world to the urgent need to discover new treatments and activities targeted to diminishing viral spread. Indeed, it has been suggested that marine-derived compounds could be a good alternative against coronaviruses [9]. SARS-CoV-2 is an enveloped, single-stranded, positive-sense RNA virus belonging to the Betacoronavirus group of the family Coronovidae [10]. Metabolites from the soft coral *Nephthea* sp. have been identified which can be used as potential SARS-CoV-2 protease inhibitors [11]. A series of cembranoid diterpenes from the genus *Sarcophyton* have been examined as SARS-CoV-2 Mpro inhibitors [12]. The compound Bislatumlide A from the same genus has been reported to remodulate the p38 MAPK signaling pathway hijacked by SARS-CoV-2 infection, thereby antagonizing its harmful effects [12]. Tuaimenal A (**1**) from the Irish deep-sea soft coral *Duva florida* was found to inhibit the main protease of SARS-CoV-2 [13].

Bioactive compounds are also being screened for novel and alternative means to combat the infectivity of established viruses that cause significant disease burden, particularly in low-income settings [14]. Human papillomaviruses (HPVs), for example, are nonenveloped small DNA tumor viruses that infect keratinocytes of the differentiating epithelium of the skin and mucosa [15], and high-risk HPV types have been identified as the etiological agent of cervical and other anogenital cancers [16]. Although highly effective prophylactic HPV vaccines are available, they do not offer protection against all cancer-associated HPV types and are often too expensive for nationwide roll-outs outside of developed countries [17]. Therefore, the search for potential drug candidates with high inhibitory activities against various HPV types is increasing in the pharmaceutical industry. Marine-derived natural bioactive compounds and their derivatives are great sources for the development of new-generation anti-HPV therapeutics, which is more effective with fewer side-effects [18]. Over the years, different marine compounds have been studied intensively for their antiviral effect, and carrageenan is in the limelight. Carrageenans are one of the major constituents of red seaweed cell walls and are mainly extracted from certain genera of red seaweeds [19,20]. The three main types of carrageenans, λ-, κ-, and ι-carrageenans, each show different inhibitory effects on different viruses, such as HPV [17], dengue virus (DENV) [21], human immunodeficiency virus (HIV) [22], and influenza A virus [23]. However, until now, no work has been published on the antiviral activity of soft coral metabolites against HPV infection.

Our previous work showed that soft corals around Mauritius Island have antimicrobial effects, and the GCMS-MS analysis indicated the presence of compounds with potential antiviral effects [6]. Therefore, our current study investigated the chemical profile of four soft corals extracts, namely, *Sinularia polydactyla*, *Cespitularia simplex*, *Lobophytum patulum*, and *Lobophytum crassum*, using LC-MS. Furthermore, metagenomic analysis was employed to identify the microbial community harboring on or in the soft corals’ tissues which might be responsible for the presence of the different biological compounds identified by LC–MS. Finally, we investigated the antiviral activities of the four soft coral extracts against SARS-CoV-2 and HPV pseudovirus infection.

## 2. Results

### 2.1. Metabolic Profiling

**Figure 1:**
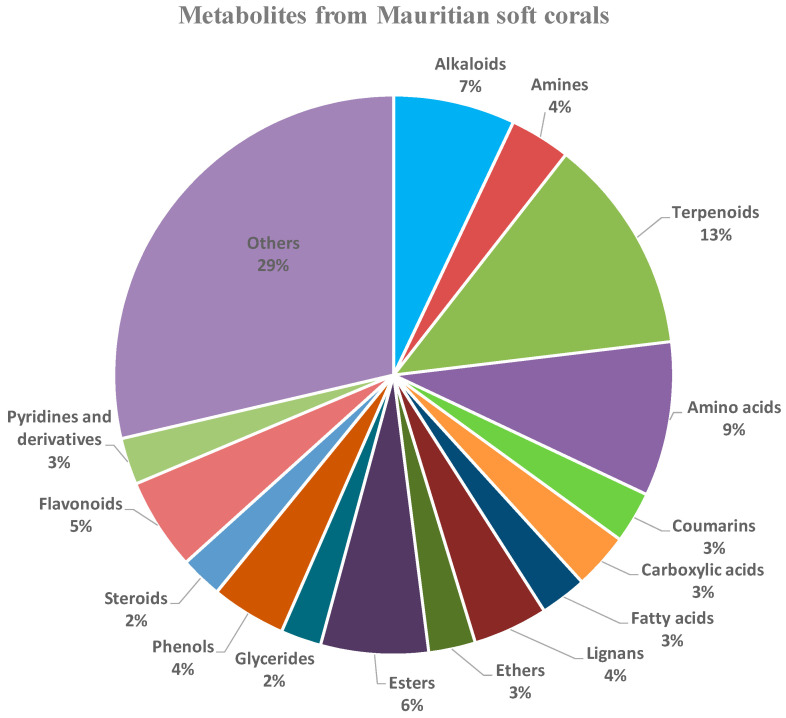
Pie chart of the classes of metabolites identified in soft coral samples. The category “Others” includes compounds such as ketones, heterocyclic aromatic compounds, and nucleosides. The pie chart was created from all the metabolites listed as Supplementary Data S1.

The identification of the metabolites from the four studied soft corals, namely, *Sinularia polydactya*, *Cespitularia simplex*, *Lobophytum patulum*, and *Lobophytum crassum*, was achieved using the LC-MS technique. Terpenoids were observed to be the most abundant class. Additionally, alkaloids, esters, flavonoids, steroids, and coumarins were also detected in the soft corals (Figure 1). LC-MS analysis identified 362 metabolites in *S. polydactyla*, 379 in *L. patulum*, 368 in *C. simplex*, and 370 in *L. crassum*. When analyzing the constituents from the different extracts, the soft coral extracts contained metabolites with molecular masses predominantly in the range *m/z* 90–500 (Supplementary Data S1). 

**Figure 2:**
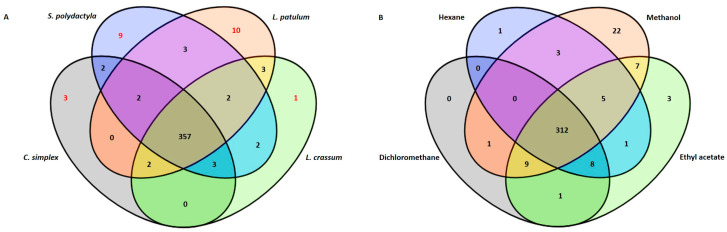
Venn diagram showing the numbers of (**A**) unique metabolites present in each soft coral, where *L. patulum* shows the highest number of unique metabolites compared to the other soft corals; (**B**) metabolites extracted using the different solvents of *L. patulum*. Methanol extracted more metabolites than any of the other solvents.

The data also revealed that the four soft corals produced nearly identical metabolites; however, the production of certain metabolites was species-specific (Table 1). Ten unique metabolites were identified from *L. patulum*, nine from *S. polydactyla*, two from *C. simplex*, and one from *L. crassum*, as illustrated in Figure 2A. Furthermore, the methanol extract of *L. patulum* yielded a higher number of compounds compared to the other solvents (Figure 2B).

Certain metabolites derived from the studied soft corals have been noted for their distinctive biological properties (Table 1). From *L. patulum*, seven of the ten unique metabolites exhibited intriguing biological properties. Specifically, 2-amino-4-hydroxypyrimidine-5-carboxylic acid is known for its antibacterial effects [24], while bruceine D demonstrated antitumor properties [25], and terbutaline was reported for its anti-inflammatory, antiarthritic effects, as well as bronchodilator properties [26,27]. Additionally, cyclopamine, eurycomalactone, and quinaldic acid were found to possess antiviral properties [28,29,30].

Out of the nine unique metabolites retrieved from *S. polydactyla*, two of them, namely, erianin and methotrexate, have been reported for their biological properties, such as antitumor, antiviral, anti-inflammatory, and immunosuppressant properties [31,32,33,34]. Additionally, ketoconazole, reported in the *C. simplex* extract only, was reported to have antifungal, antiviral, anticancer, and anti-inflammatory properties [35,36,37,38]. 

Interestingly, all soft corals contained metabolites with previously reported antiviral activities, as shown in Table 2.

**Table 1:** Unique metabolites present in the soft corals and their biological properties.

Soft Corals	Metabolites Name	Average Rt (min)	Biological Properties	References
***L. patulum***	2-amino-4-hydroxypyrimidine-5-carboxylic acid	4.586	Antibacterial	[24]
	Cyclopamine	10.841	Antiviral (RSV, BRSV); Antitumor	[28,39,40]
	Bruceine D	11.117	Antitumor	[25]
	5-(hydroxymethyl)pyrimidine-2,4-diol	9.145	-	-
	2-(3,5-Dimethyl-7-oxo-7H-furo[3,2-g]chromen-6-yl)-N-[3-(2-oxo-1-pyrrolidinyl)propyl]acetamide	11.771	-	-
	Eurycomalactone	11.27	Antiviral (HCoV-OC43 and SARS-CoV-2 strains); Anticancer	[29,41]
	Neosolaniol	11.223	-	-
	Quinaldic acid	11.117	Antiviral (influenza A/H5N1); Antibacterial; Anticancer;	[30,42,43]
	Terbutaline	9.151	Bronchodilator; Anti-inflammatory; Antiarthritic	[26,27]
	Tetrahydrozoline HCl	11.323	Topical nasal and conjunctival decongestant	[44]
***S. polydactyla***	2-((6-((6,7-dimethoxy-3,4-dihydroisoquinolin-2(1H)-yl)methyl)-4-oxo-4H-pyran-3-yl)oxy)-N-(3,4-dimethoxyphenethyl)acetamide	13.187	-	-
	2-(3,4-dimethoxyphenyl)-7-methoxy-4H-chromen-4-one	12.817	-	-
	24-epimakisterone A	11.834	-	-
	7-amino-flunitrazepam	13.568	-	-
	Erianin	13.521	Antitumor; Antiviral (Human enterovirus 68); Anti-inflammation	[31,32,33,34]
	Linderane	12.927	-	-
	Pregn-4-ene-3,20-dione	12.254	-	-
	Methotrexate	0.674	Anticancer; Immunosuppressant	[45,46]
***C. simplex***	Ketoconazole	0.39	Antifungal; Antiviral (HSV-1/HSV-2); Anticancer; Anti-inflammatory	[35,36,37,38]
***L. crassum***	Methyl 3-(6-((4-formylpiperazin-1-yl)methyl)-3-hydroxy-4-oxo-4H-pyran-2-yl)-3-(4-((1-methyl-1H-imidazol-2-yl)methoxy)phenyl)propanoate	6.329	-	-

### 2.2. Associated Bacterial Communities

**Figure 3:**
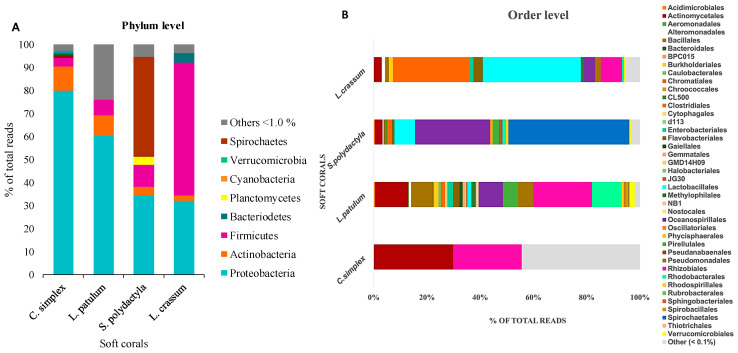
Stacked bar chart showing relative abundance of (**A**) bacterial phyla obtained by sequencing of Mauritian soft corals. (**B**) The relative abundance is shown as in A, but specified by the order taxonomic level. Orders that constituted to less than 0.1% of the community were grouped under other.

At the taxonomic classification level of phyla, operational taxonomic units (OTUs) affiliated with the Proteobacteria phylum exhibited a predominant presence within the two investigated soft coral species, *C. simplex* and *L. patulum*, constituting 79% and 60% of their respective microbiomes (Figure 3A). Conversely, in *S. polydactyla* and *L. crassum*, Proteobacteria accounted for only 30% and 27%, respectively. Spirochaetes emerged as the most abundant phylum within *S. polydactyla*, representing 45% of the microbial community. In *L. crassum*, the Firmicutes phylum dominated with an approximate representation of 70%. Cyanobacteria were also present in the soft corals, but to a lower extent, hovering at around 10% in *C. simplex* and *L. patulum*, and approximately 5% in *S. polydactyla and L. crassum.*

The OTUs assigned to the order level (Figure 3B) revealed noteworthy insights. Within *L. crassum*, the prevalent order was Lactobacillales, accounting for approximately 20% of the composition, closely followed by Clostridiales at 15%. In *S. polydactyla*, Spirochaetales dominated the bacterial order composition with a substantial 40%, although its prevalence in other soft coral species was notably lower. Oceanospirillales was ranked as the second most abundant order in *S. polydactyla*, comprising 25% of the community, while constituting less than 10% in the remaining soft coral species. *L. patulum*, in comparison to the others, exhibited a broader spectrum of bacterial orders, with the order Rhizobiales exhibiting the highest abundance of 18%. Interestingly, within *C. simplex*, only two orders, Actinomycetales and Rhizobiales, were substantially abundant, each accounting for less than 30%, while most of the other bacterial orders formed part of the ‘Others’ category, as they had an abundance of less than 0.1%. A detailed report of taxonomic rank based on normalized proportion is provided as Supplementary Data S2–S5.

**Figure 4:**
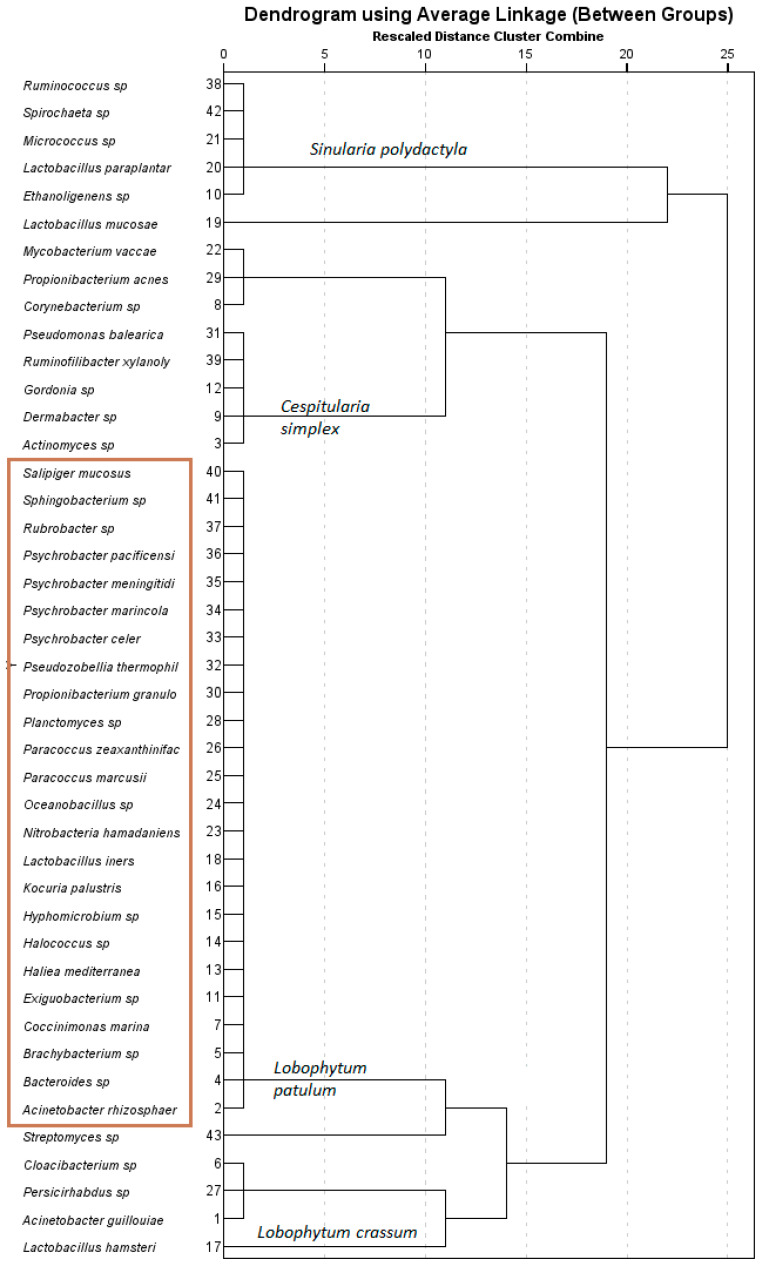
Dendrogram based on the average linkage (between-group) method using the different bacterial species associated with the different soft corals collected. Species that constituted less than 0.1% of the community were not included in the dendrogram. The species in the red box are unique bacterial species found in *L. patulum*.

Average linkage analysis revealed the presence of a higher number of unique bacterial species in *L. patulum* compared to the other soft corals (Figure 4). *L. patulum* exhibited symbiotic relationships with several noteworthy bacterial taxa, including *Salipiger mucosus*, *Psychrobacter marincola*, *Psychrobacter* sp., *Propionibacterium* sp., *Paracoccus marcussi*, *Kocuria palustris*, *Exiguobacterium* sp., and *Brachybacterium* sp. These bacterial species have been documented as prolific producers of metabolites bearing substantial biological importance (Table 3). According to published data, the bacteria *Paracoccus marcussi*, *Exiguobacterium* sp., and *Brachybacterium* sp. produce metabolites which have antimicrobial properties [59,60,61], while *Salipiger mucosus* and *Propionibacterium* sp. have been observed to yield compounds exhibiting antiviral activity [62,63]. Furthermore, biological compounds isolated from *Psychrobacter marincola*, *Psychrobacter* sp., and *Mycobacterium vaccae* demonstrated antitumor and anticancer properties [64,65,66].

**Table 3:** Overview of the more abundant associated bacteria (genus/species level) from Mauritian soft corals, and their biological compounds and properties, using published references (S.c = *S. polydactyla*; C.s = *C. simplex*; L.p = *L. patulum*; L.c = *L. crassum*).

Soft Corals	Associated Bacteria	Biological Compounds	Biological Properties	References
**L.p**	*Salipiger mucosus*	Exopolysaccharides	Antiviral; Antiangiogenic	[62]
	*Psychrobacter marincola*	Capsular polysaccharides	Antitumor	[64]
	*Psychrobacter* sp.	Bile acid derivative	Antibacterial, Cytotoxic to tumor cell lines	[65]
	*Propionibacterium* sp.	Propionic acid	Antiviral	[63]
	*Paracoccus marcussi*	-	Antibacterial	[59]
	*Kocuria palustris*	Alkaloids	Antifungal	[67]
	*Exiguobacterium* sp.	-	Antibacterial	[60]
	*Brachybacterium* sp.	Exopolysaccharides	Antibacterial	[61]
**L.p/C.s**	*Mycobacterium vaccae*	-	Cancer treatment	[66]
**C.s**	*Gordonia* sp.	-	Antimicrobial	[68]
**C.s**	*Dermobacter* sp.	Imidazolium compound	Antibacterial	[69]
**C.s**	*Actinomyces* sp.	Tetradecanoic acid, pentadecanoic acid, n-hexadecanoic acid	Antifungal; Antimicrobial	[70,71]
**L.p/S.p**	*Streptomyces* sp.	Polyketides, alkaloids and terpenoids Strepchloritides A and B, Polyketone, 9(10H)-acridanone	Antiviral (H1N1, SARS-CoV-2, white spot syndrome virus (WSSV))	[72,73,74,75]
**L.p/S.p/L.c/C.s**	*Lactobacillus* sp.	Intercellular polysaccharides/exocellular polysaccharides	Prebiotics with bifidogenic effect	[76]

### 2.3. Cell-Viability Analysis

As mentioned above, the four soft corals, *Sinularia polydactyla*, *Cespitularia simplex*, *Lobophytum patulum*, and *Lobophytum crassum*, were extracted sequentially using four different solvents, namely, hexane, dichloromethane, methanol, and ethyl acetate. The LC–MS data revealed the presence of biologically important compounds in the four soft corals, particularly in *L. patulum*, which had a heightened number of unique compounds compared to the others (Figure 2A) and showed the most efficient extraction when using methanol (Figure 2B).

**Figure 5:**
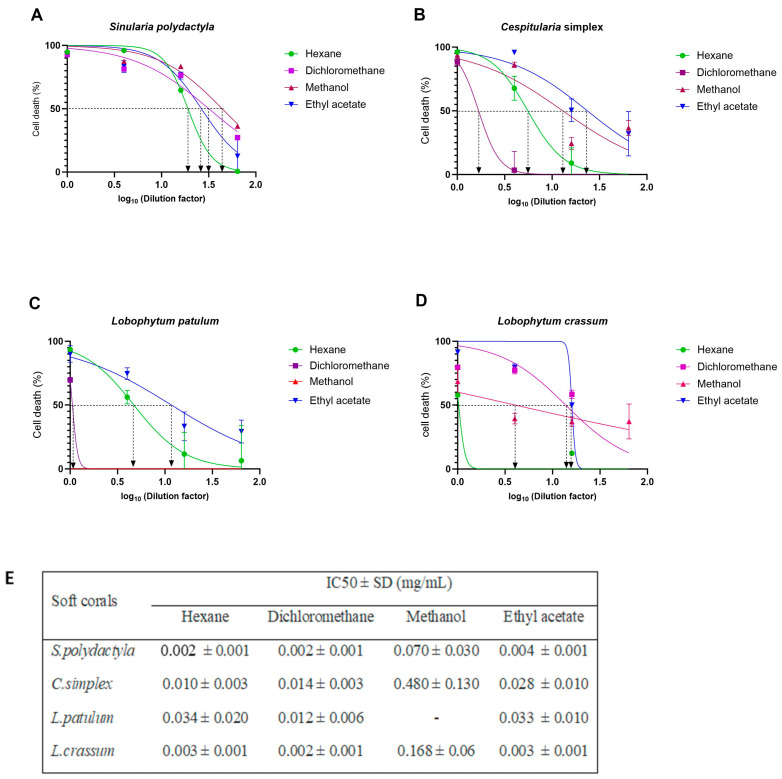
Cell death of HEK293T-ACE2 cells and IC_50_ concentration of soft coral extracts measured by MTT assay. The normalized percentage cell-death values were plotted against the logarithm of the dilution factors. HEK293T-ACE2 cells were exposed to different concentrations of (**A**) *S. polydactyla* extracts, (**B**) *C. simplex* extracts, (**C**) *L. patulum* extracts, and (**D**) *L. crassum* extracts. (**E**) Summary of all IC_50_ values for all tested soft coral extracts against HEK293T–ACE2 cells. The IC_50_ was determined by three independent experiments using nonlinear regression analysis in GraphPad Prism Software® v10.0.2.

The cytotoxicity of each extract was evaluated on HEK293T-ACE2 cells using four-fold serial dilutions, and the IC_50_ value of each extract was determined (summarized in Figure 5E). Extracts from the soft corals *S. polydactyla* and *L. crassum* exhibited IC_50_ values ranging from 0.002 to 0.168 mg/mL, depending on the extraction method. *C. simplex* and *L. patulum* extracts showed IC_50_ values in the range from 0.010 to 0.480 mg/mL. Furthermore, the methanol extract of all soft corals displayed higher IC_50_ values, ranging from 0.07 to 0.480 mg/mL, than other extraction methods. It is worth noting that no IC_50_ value was determined for the methanol extract of *L. patulum*, indicating its nontoxicity to the cells.

**Figure 6:**
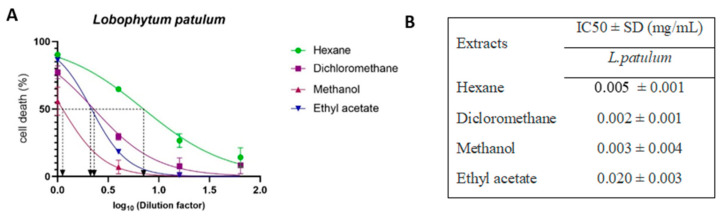
Cell death of HaCaT cells and IC_50_ concentrations of *L. patulum* extracts measured by MTT assay. (**A**) HaCaT cells were exposed to different concentrations of the different *L. patulum* extracts. The normalized percentage cell-death values were plotted against the logarithm of the dilution factors. (**B**) Summary of IC_50_ values for *L. patulum* extracts against HaCaT cells as determined by nonlinear regression analysis using GraphPad Prism Software® v10.0.2.

*L. patulum* extracts, which showed to be least toxic to HEK293T-ACE 2 cells (Figure 5), were further assessed for their cytotoxicity on HaCaT cells (Figure 6). Interestingly, *L. patulum* was found to be more toxic to HaCaT cells than to HEK293T-ACE2 cells, with IC_50_ values ranging between 0.002 and 0.020 mg/mL for HaCaT cells compared to IC_50_ values between 0.012 and 0.034 mg/mL (and even not detectable) for HEK293T-ACE2 cells. Hexane, DCM, and methanol extracts showed the highest toxic effects on HaCaT cells, with IC_50_ values ranging from 0.002 to 0.005 mg/mL. Ethyl acetate extract, however, had a relatively lower toxicity towards HaCaT cells, with an IC_50_ value of 0.020 mg/mL. 

Using these IC_50_ values, all the different extracts of each soft coral were investigated for their anti-SARS-CoV-2 activities, while only the *L. patulum* extracts were further investigated for their anti-HPV activities.

### 2.4. Antiviral Activity

**Figure 7:**
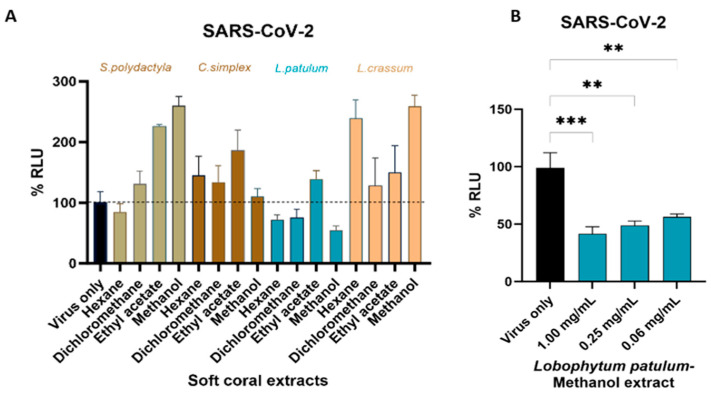
Antiviral activity of all soft coral extracts against SARS-CoV-2-spike-pseudotyped viruses. (**A**) The % relative firefly luciferase activity (RLU) was determined after treating HEK293T-ACE2 cells with the ½ IC_50_ concentrations of the individual extracts for 24 h, followed by infection with SARS-CoV-2 pseudovirions for 48 h. (**B**) The % RLU of cells treated with the methanol extract of *L. patulum* at the indicated concentrations followed by infection with SARS-CoV-2-spike-pseudotyped viruses. The mock-treated sample was set at 100%. Data are expressed as the mean ± S.D. of three independent experiments. Luciferase data were normalized to cell viability derived from a parallel plate with identical set-up. ** *p* < 0.01, *** *p* < 0.001.

Based on the cytotoxic activity of the fractions of the four soft corals, the SARS-CoV-2-spike-pseudotyped virus infection assays were next performed to evaluate if the soft coral extracts inhibited viral entry in HEK293T-ACE2 cells. Under our experimental conditions, we identified *Lobophytum patulum* extracts to exhibit some antiviral activity against SARS-CoV-2, with the hexane and DCM extracts inhibiting viral entry by 27.8 ± 7.6% and 24.1 ± 13.1%, respectively. In contrast, the other soft coral extracts were found to increase the infectivity of the pseudotyped virus, though not significantly (Figure 7A). Being nontoxic to the cells, and having some antiviral activity, three different concentrations of the *L. patulum* methanol extract obtained by four-fold serial dilution were then used to further evaluate the inhibition of viral entry into HEK293T-ACE2 cells (Figure 7B). The highest viral entry inhibition was observed at a concentration of 1.00 mg/mL, resulting in 58.5 ± 6.1% inhibition, with decreasing concentrations leading to decreased inhibition of viral entry.

**Figure 8:**
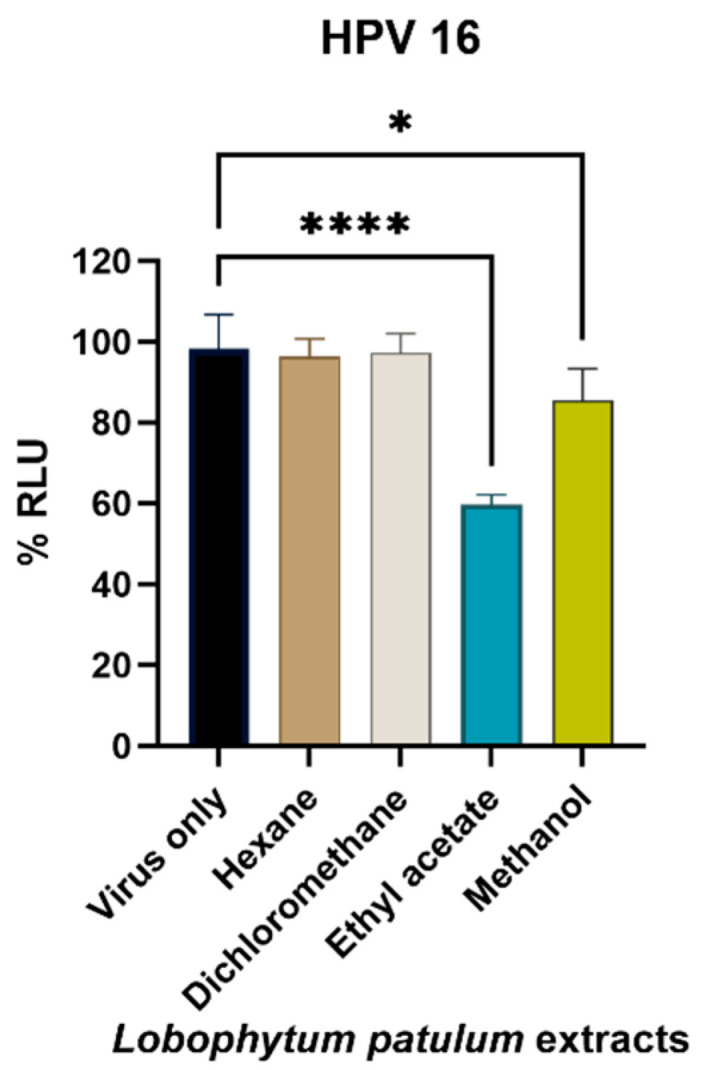
Antiviral activity of all *L. patulum* extracts against HPV16-PsVs. HaCaT cells were treated at ½ IC_50_ concentration of the *L. patulum* extracts for 24 h, followed by infection with HPV16-PsVs for 48 h. Relative Gaussia luciferase activity (RLU) was determined by setting the mock-treated sample at 100%. Data are expressed as the mean ± S.D. of three independent experiments. Luciferase data were normalized to cell viability derived from a parallel plate with identical set-up. * *p* < 0.1; **** *p* < 0.0001.

Following the results obtained for the inhibition of SARS-CoV-2 pseudovirus entry into HEK293T-ACE-2 cells, the selected soft coral for the further evaluation of antiviral activities was *L. patulum*. The HPV16 pseudovirus entry into HaCaT cells (Figure 8) was further investigated, and the *L. patulum* ethyl acetate extract was found to have the capacity to inhibit viral infection by 40.1 ± 3.9% at a concentration of 0.04 mg/mL, followed by the methanol extract, which inhibited infectivity by 14.5 ± 7.1%.

In summary, the extracts derived from *L. patulum*, characterized by a heightened abundance of metabolites and a greater spectrum of bacterial diversity, demonstrated a noteworthy capacity for eliciting antiviral effects against both SARS-CoV-2 and HPV16 pseudoviruses.

## 3. Discussion

The lack of calcium carbonate skeletons in soft corals make them more susceptible, and hence they rely strongly on chemical defense for their protection from predators in a highly competitive marine environment [77]. The protective/defensive metabolites found in soft corals are of pharmaceutical interest, as they exhibit distinct bioactivities, such as antimicrobial, antiviral, anti-inflammatory, and anticancer activities [78]. Existing and emerging viral infections pose a significant threat to global populations, as demonstrated by the ongoing challenges posed by viruses like HIV, influenza, SARS-CoV-2, HPV, and many others [79]. In response to the rising demand for the identification of novel antiviral compounds in the ongoing battle against existing and emerging viral diseases, the present study examined the in vitro antiviral activities of four Mauritian soft coral extracts.

The antiviral potential of the soft coral extracts was assessed against SARS-CoV-2 and HPV16 pseudovirus infections. Notably, the species *L. patulum* exhibited remarkable antiviral efficacy against both pseudoviruses, thereby representing the first instance of such activity reported for this species. While information concerning the chemical composition and antiviral attributes of *L. patulum* remains absent in the existing literature, prior research has highlighted the antiviral properties of various other *Lobophytum* species [20,80]. Interestingly, the genus *Lobophytum* has been recognized as a prolific source of secondary metabolites, characterized by diverse biological functionalities encompassing antibacterial, anticancer, and anti-inflammatory properties [81,82,83]. 

The different antiviral potentials observed for the different soft coral species and extraction methods could be a result of the quantitative and qualitative abundance of metabolites. In the case of *L. patulum*, ten unique metabolites were discerned within the crude extracts, out of which some have previously been acknowledged for their antiviral properties against other groups of viruses. For example, a study by Choonong et al. [29] revealed potent antiviral activities of eurycomalactone, a quassinoid compound, against both HCoV-OC43 and SARS-CoV-2 [29], with low IC_50_ values ranging from 0.32 to 0.51 μM. In another study, Bailly et al. (2016) demonstrated that cyclopamine exhibited inhibitory effects on the human respiratory syncytial virus (hRSV) via a unique Smo-independent mechanism [28]. Furthermore, the antiviral potential of derivatives of quinaldic acid against the influenza A/H5N1 virus has been reported [30]. It is important to note, however, that the observed antiviral effects of the *L. patulum* extracts found in our study cannot be solely attributed to the presence of unique compounds, but are more likely due to combined synergistic effects with other compounds found in this species. Lobane diterpenoids, for example, which have been recognized as one of the most prolific components of the genus *Lobophytum*, demonstrated remarkable pharmacological potential as well [84]. 

Interestingly, all four studied soft corals contained metabolites with previously reported antiviral effects, namely catechin, 2-aminobenzothiazole, lysine, nylidrin, quinoxaline, and tenofovir. Catechin was reported to have an entry-inhibitory role against SARS-CoV-2 through binding to the S1 domain of the spike protein, thereby effectively blocking its interaction with the ACE2 receptor and preventing viral infection [50,85]. A derivative of catechin demonstrated growth-inhibitory potential in four human papillomavirus-infected tumor cell lines [52]. Furthermore, studies on the mechanism of action of lysine demonstrated that it may disrupt SARS-CoV-2 virus uncoating instead of affecting virus attachment and endosomal acidification [54]. While our study only focused on the effect of the soft coral extracts on viral entry, these reports demonstrate the potential for further research into targeting downstream steps in the viral lifecycle and virus-associated pathogenesis by using soft coral extracts. In addition to the characterization of the bioactive compounds in the four soft coral species, our study also analyzed the microbial structure associated with the soft corals, as coral-associated bacteria have been recognized as the true sources of biologically active compounds in corals [86,87]. Indeed, some compounds isolated from soft corals have great similarities to the metabolites produced by their symbiotic bacteria [87]. To our knowledge, our study represents the first characterization of Mauritian soft coral-associated bacterial communities based on operational taxonomic units (OTUs). These were categorized into major phyla, including Proteobacteria, Spirochaetes, Firmicutes, and Cyanobacteria, although we experienced limitations in precisely matching all bacterial sequences to established reference sequences of biologically significant bacteria. Nevertheless, our findings did reveal the presence of certain associated bacterial communities that have previously been documented for their ability to produce biologically active metabolites. For example, the isolation of 9(10H)-acridanone from the genus Streptomyces has been reported, which has antiviral activities against white spot syndrome virus (WSSV) [75]. Interestingly, this compound was also identified in the soft corals from this study. 

It should be noted that not only complex microbial communities associated with soft corals could be responsible for metabolite production. Environmental conditions, such as light intensity, pH, water temperature, nutrient availability, as well as the water quality, can impact the physiology of marine organisms, altering the production of secondary metabolites [88]. However, these data were not assessed in this study. 

The development of effective antiviral drugs is rather challenging, primarily due to the intricate task of only targeting the virus without affecting the host cells [89]. Mechanisms of action of antiviral drugs include enhancing cellular resistance to viral infections, such as inhibiting viral entry, intracellular trafficking, and deproteinization within the cell, as well as antimetabolites that cause inhibition of viral replication [89]. It is noteworthy that the present study focused only on investigating antiviral activities against viral entry by using pseudovirions. The observed results suggest that the unique compounds, either in isolation or in combination with the common compounds identified in *L. patulum*, exhibit the potential to act as inhibitors of viral entry into cells. More in-depth work is required to decipher the underlying mechanisms of action in order to identify potential novel antiviral drug targets.

## 4. Materials and Methods

### 4.1. Soft Coral Materials

**Figure 9:**
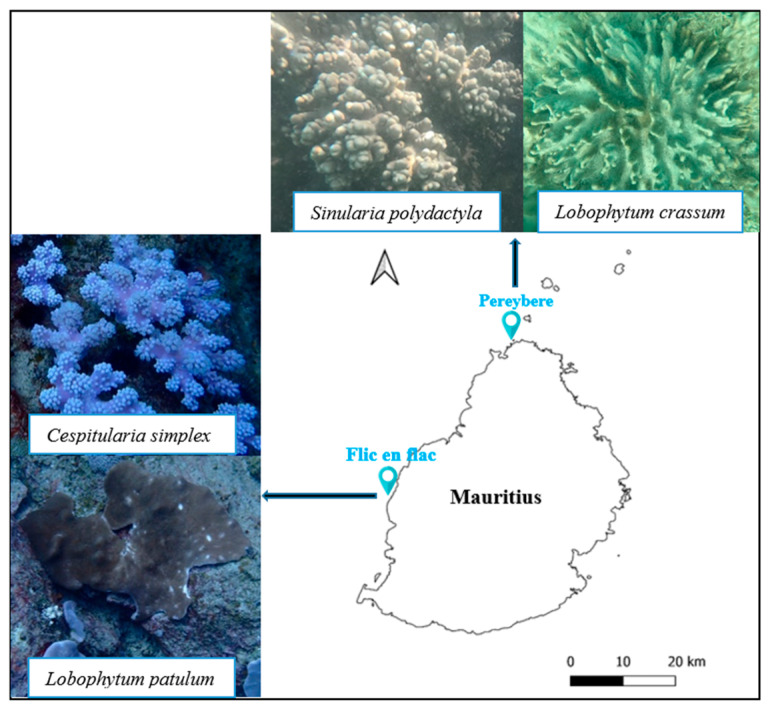
Map showing selected sampling sites around Mauritius and the soft coral species collected from the sampling sites used in this study.

Four soft corals, namely, *Sinularia polydactyla*, *Cespitularia simplex*, *Lobophytum patulum*, and *Lobophytum crassum*, were collected from Pereybere and Flic en Flac (Figure 9). The samples from Flic en Flac were collected at a depth of 15 m by scuba diving and the soft corals from Pereybere were collected at a depth of 2 m by snorkeling. The fresh samples were transported in seawater to the laboratory, where they were cleaned and frozen at −80 °C before freeze-drying. Taxonomic identification of the soft corals was confirmed using the mitochondrial-protein-coding primers ND42599F and Mut-3458R [90], and submitted to GenBank NCBI. *S. polydactyla* was given the accession number OQ616755, *L. patulum* OR513793, *C. simplex* OR538714, and *L. crassum* OR548245.

#### 4.1.1. Preparation of Extracts

All samples were freeze-dried and shipped to the International Centre for Genetic Engineering and Biotechnology (ICGEB) for further processing. Each dried soft coral sample (20–130 g) was sequentially extracted with different solvents in the increasing polarity order [91]. Briefly, each soft coral was macerated separately in 150 mL hexane with intermittent shaking for 24 h. Then, they were first filtered with muslin cloth and then through Whatman n_o_ 1 filter paper. The resulting residue was air-dried and further extracted with dichloromethane (DCM), followed by ethyl acetate, and then methanol, similar to the procedure carried out for the hexane extraction. Finally, the solvent was removed from each filtrate using a rotary evaporator (Rotavapor® R-300) under reduced pressure and low temperature. The yield of each extract was weighed and stored at −20 °C. For the antiviral assay, each extract (15 to 90 mg) was dissolved in 0.5–1.0 mL of dimethyl sulfoxide (DMSO), sterilized using a 0.44 μm syringe filter, and stored in 1.5 mL light-sensitive vials at −80 °C. The percentage yield, as well as the concentration of each soft coral extract used for the antiviral assays, was calculated using the Equations (1) and (2) respectively and the data was provided in Tables S1 and S2:

(1) $$\%\;yield=\frac{Mass\;of\;extract\;(g)}{Mass\;of\;sample\;used\;(g)}\times 100$$

(2) $$Concentration\;(mg/mL)=\frac{Mass\;of\;extract\;(mg)}{Volume\;of\;solvent\;used\;to\;dissolve\;the\;extracts\;(mL)}\;$$

#### 4.1.2. Metabolic Profiling 

Metabolic profiling of the different soft coral extracts was performed in the Division of Chemical & Systems Biology, University of Cape Town. A Thermo Fisher Scientific Ultimate 3000 nano-LC system coupled to a Q Exactive Plus Orbitrap mass spectrometer, following published procedures [92], was employed. Prior to injection onto the liquid chromatography (LC) system, samples were centrifuged at 3500× *g* for 5 min to remove insoluble debris. Chromatographic separation was performed on the Hypersil GOLD C18 (100 mm × 2.1 mm, 3 μm; Thermo Scientific, Waltham, MA, USA) column. The mobile phase used for chromatographic separation was composed of acetonitrile (solvent B) and water (containing 0.1% formic acid, solvent A). The flow rate was 0.3 mL/min.

Eluted compounds were directly introduced into the mass spectrometer. Optimal parameters were as follows: probe heater temperature, 350 °C; spray voltage, 3.5 kV for the positive- and negative-ion modes; sheath gas, 35 arb; auxiliary gas, 10 arb. Capillary temperature was set at 320 °C and S-lens was 50 V. Full-scan MS data were generated across a mass range of 100–1500 Da. The stepped normalized collision energy setting was 25 and 30 eV. Data were acquired by Xcalibur software version 3.0. All analytes were identified using their elemental composition, accurate mass measurement, elution order, fragmentation behavior, fragmentation pattern of the standard compound, and comparison with reliable data in the compounds database [93]. The feature table of compounds within the extracts was generated through the process of uploading and converting the raw data into MSDIAL Version 4.80. This table encompassed parameters, including retention time, precursor mass-to-charge ratio (*m*/*z*), adduct ion type, and the mass spectrometry (MS) type. During the MSDIAL alignment running, an inner authentic standards database “MSMS_Public_EXP_Pos_VS17” from the MSDIAL platform was matched based on precursor mass and MS/MS similarity. By matching precursor masses and assessing MS/MS similarity, MS-DIAL suggested potential identifications of the metabolites present in our samples. The feature table was then automatically compared with different databases, such as Drug Bank, PubChem, NANPDB, COCONUT, KNApASck, ChEBI, and UNPD [94].

### 4.2. Metagenomics Analysis

Frozen samples were thawed, and tissues were broken down into small pieces and macerated at room temperature. Total microbial community DNA was extracted from macerated tissues using a ZymoBIOMICSTM DNA Miniprep Kit (Zymo research, Irvine, CA, USA). About 15 mg of macerated tissues was transferred to bead-beating tubes and vortexed horizontally at maximum speed for 10 min at room temperature. DNA was extracted, precipitated, and purified according to the manufacturer’s instructions. The purity and concentration of the extracted DNA were checked using a NanoDrop Spectrophotometer (Thermo Fisher Scientific, Waltham, MA, USA). The DNA was then stored at −20 °C until PCR amplification. The V3–V4 hypervariable regions of bacterial 16S rRNA genes were amplified using the universal primer pair 341F (CCTACGGGNGGCWGCAG) and 805R (GACTACHVGGGTATCTAATCC) [95,96]. The primers were synthesized with a specific Illumina overhang adapter. The PCR amplification and sequencing was conducted at Inqaba (Pretoria, South Africa) according to the Illumina 16S Metagenomic Sequencing Library protocols (www.illumina.com (accessed on 12 May 2023)). The bioinformatics analysis was conducted by Inqaba (Pretoria, South Africa). Reads were processed through usearch (https://drive5.com/usearch (accessed on 12 May 2023)) and taxonomic information was determined based on the Ribosomal Database Project’s (http://rdp.cme.msu.edu/index.jsp (accessed on 12 May 2023)) 16S database v16, or in the case of ITS1F, the RDP ITS V2 database. Operational taxonomic units (OTUs) contributing less than 0.1% of the total data were excluded.

### 4.3. Bioassays

#### 4.3.1. Cell Culture

HaCaT and HEK293T/17 cells (American Type Culture Collection, Manassas, VA, USA), HEK293-TT cells [97,98], as well as HEK293T cells stably expressing the ACE2 receptor (HEK293T-ACE2) [99], were maintained in high-glucose Dulbecco’s Modified Eagle Medium (DMEM) supplemented with 1% penicillin/streptomycin and 10% fetal bovine serum (Thermo Fisher Scientific, Waltham, MA, USA) at 37 °C in a humidified atmosphere and 5% CO_2_. Fresh culture medium was supplied every other day. HEK293T–ACE2 cells were grown in the presence of 3 µg/mL puromycin (Thermo Fisher) to maintain ACE2 expression.

#### 4.3.2. Preparation of SARS-CoV-2 and HPV16 Pseudovirions

Single-cycle infectious SARS-CoV-2 pseudovirions based on the HIV backbone expressing the SARS-CoV-2 spike protein and a firefly luciferase reporter were produced in HEK-293T/17 cells by cotransfection of plasmids pNL4–3.Luc.R-.E- (NIH AIDS Reagent Program (#3418), Germantown, MD, USA) and pcDNA3.3-SARS-CoV2-spike Δ18 [100]. HEK-293T/17 cell-culture supernatants containing the virions were harvested 3 days post-transfection, filtered through 0.45 micron filters, and stored at −80 °C. Infectivity was tested on HEK293T–ACE2 cells using serial dilutions, and luciferase activity 3 days postinfection was measured using a GloMax^®^ Explorer Multimode Microplate Reader (Promega Biosciences, San Luis Obispo, CA, USA) together with the Luciferase assay system (Promega). Virus preparations that yielded RLU values between 50,000 and 200,000 were selected for further infection experiments.

HPV16-PsVs encapsidating the secreted Gaussia luciferase reporter gene plasmid pCMV-GLuc2 (New England Biolabs, Ipswich, MA, USA) were produced in HEK-293TT cells by cotransfection with the plasmid pXULL, which encodes codon-optimized HPV16 L1 and L2, following published procedures [98,101]. Virions were purified by CsCl density gradient centrifugation, as described [102], and protein concentration determined by BCA assay (Thermo Fisher). Quality controls of the pseudovirus preparations were performed as described [98,101]. For infection experiments, a final protein concentration of 0.1 µg/µL was used.

#### 4.3.3. Cytotoxicity Assay

Cytotoxicity assays were carried out to determine noncytotoxic concentrations of the soft coral extracts to be used in the antiviral assays. HEK293T–ACE2 or HaCaT cells, respectively, were seeded at a density of 1 × 10^5^ cells/well in 96-well microplates and incubated for 24 h at 37 °C in a humidified incubator with 5% CO_2_. Thereafter, 100 µL of each extract at four different concentrations, which were obtained by four-fold serial dilution in DMEM, were added. The stock concentrations of each soft coral extract are given in Supplementary Data S3. The control wells contained cells without any extract treatment. The microplates were incubated at 37 °C in a humidified incubator with 5% CO_2_ for 48 h. 

Cell viability was determined by the MTT (3-[4,5-dimethylthiazol-2-yl]-2,5 diphenyl tetrazolium bromide) assay following published procedures [103]. Optical density was measured spectrophotometrically (GloMax^®^ Explorer, Promega) at 590 nm. The experiments were conducted in triplicate and the 50% inhibitory concentration (IC_50_) was calculated by nonlinear regression analysis using GraphPad Prism Software^®^ v10.0.2. For the antiviral assays, the ½ IC_50_ concentrations of each extract were used.

#### 4.3.4. Inhibiting SARS-CoV-2 Pseudovirus Entry into HEK293T–ACE2 Cells

The ACE2-overexpressing HEK293T cells were seeded at a density of 1 × 10^5^ cells/well in 96-well microplates. After 24 h, medium was exchanged with fresh medium containing ½ IC_50_ concentrations of the soft coral extracts, as indicated. Each concentration was tested in triplicate, and at least six nontreated control wells were included in the assay. After an incubation period of another 24 h, the medium was removed and replaced by 100 µL of virus-containing medium. The plates were incubated at 37 °C in a 5% CO_2_ humidified incubator for 48 h. Firefly luciferase RLU was then assayed using the Promega GloMax Explorer together with the Luciferase assay system kit (Promega) and normalized to cell viability. The percentage inhibition of each sample was calculated using the Equation (3).

(3) $$\%\;cell\;inhibition=\frac{A_{c}-A_{t}}{A_{c}}\times 100$$

where A_t_ = the normalized luminescence value of the test compound and A_c_ = normalized luminescence value of the control.

#### 4.3.5. Inhibiting HPV Entry in HaCaT Cells

HaCaT cells were seeded at a density of 1 × 10^5^ cells/well in 96-well microplates. After 24 h, the medium was exchanged with fresh medium containing ½ IC_50_ concentration of *L. patulum* extracts. After 24 h incubation, the medium was removed and replaced by 100 µL of fresh medium containing HPV16 pseudovirions. The plates were incubated at 37 °C in a 5% CO_2_ humidified incubator for 48 h, after which the Gaussia luciferase RLU was assayed using the Promega GloMax Explorer together with the Gaussia Luciferase Assay Kit (New England Biolabs, MA, USA). The percentage inhibition was determined according to the luciferase activity normalized to the cell viability assay.

#### 4.3.6. Data Analysis

To analyze the bacterial cohort data, the identified bacterial species were scored as a data matrix (0–1). The matrix data were ultimately used to generate a dendrogram using the SPSS Statistics 29.0 program. The option Average Linkage (Between Groups) was selected to generate the phylogenetic tree. Microsoft Excel and Graph Pad Prism (Version 10.0.2) software were used to calculate the minimum inhibitory concentration (IC_50_) of the different extracts. The percentages of cell viability at different extract concentrations were analyzed by one-way analysis of variance (ANOVA) using Graph Pad Prism (Version 10.0.2) software. The IC_50_ values were calculated from linear regression analysis.

## 5. Conclusions

Overall, this study revealed that *L. patulum* demarcates itself from the other collected soft corals due to its plethora of distinctive metabolites, a greater diversity of associated bacterial communities, and broader and more potent antiviral activities. The intricate microenvironment within soft corals presents a challenge to identify the precise nature and origin of the antiviral compounds. A comprehensive investigation of *L. patulum* to elucidate the specific bioactive compounds for downstream synthesis and assessment, either alone or in combination, holds promise as a viable avenue for the potential discovery of pharmaceutical agents with antiviral properties. Consequently, this study is considered as an important preliminary work to study the mechanisms of actions underlying the antiviral activities of Mauritian soft corals.

## Figures and Tables

**Table 2:** Soft coral metabolites detected in all four species with potential antiviral activities.

Soft Corals	Metabolite Name	Antiviral Activities	References
***S. polydactyla*, ** ***C. simplex*, ** ***L. patulum*, ** ***L. crassum***	Catechin	Human immunodeficiency virus; Herpes simplex virus; Influenza virus; Hepatitis B and C virus; SARS-CoV-2; Human papilloma virus	[47,48,49,50,51,52]
	2-Aminobenzothiazole	Hepatitis C virus	[53]
	Lysine	SARS-CoV-2; Influenza A virus	[54]
	Nylidrin	Influenza A virus	[55]
	Quinoxaline	Herpes simplex virus	[56]
	Tenofovir	SARS-CoV-2; Herpes simplex virus; Human immunodeficiency virus	[57,58]

## Data Availability

The data presented in this study are available on request from the corresponding author.

## Supplementary Materials

The following supporting information can be downloaded at: https://www.mdpi.com/article/10.3390/md21110574/s1, Supplementary Data S1: Metabolites identified from the four soft corals; Supplementary Data S2: Bioinformatics data showing all the bacterial communities present in *C. simplex*; Supplementary Data S3: Bioinformatics data showing all the bacterial communities present in *L. patulum*; Supplementary Data S4: Bioinformatics data showing all the bacterial communities present in *S. polydactyla;* Supplementary Data S5: Bioinformatics data showing all the bacterial communities present in *L. crassum*; Table S1: % yield of each soft coral; Table S2: Crude concentration of each soft coral used.
