# Supplementary material for: Metabolic Profiling, Antiviral Activity and the Microbiome of Some Mauritian Soft Corals
Source: Mar Drugs. 2023 Oct 31;21(11):574. doi: 10.3390/md21110574 (PMC10672535; doi:10.3390/md21110574)
Supplement: Supplementary file 1 [file marinedrugs-21-00574-s001.zip › Supplementary data S3 - L.patulum.pdf]

inqaba biotec™

Africa's Genomics Company

inqaba biotec metagenomic report

Sample Information

Index:	S6
Sample Name:	FF7
Run Name:	230508
Report Date:	Thu May 11 08:39:05 2023

This report contains the summarized metagenomic analysis of 16s/ITS1F gene sequencing. Samples were sequenced on an illumina system (www.illumina.com). Reads were processed through usearch (https://drive5.com/usearch) and taxonomic information was determined based on the Ribosomal Database Project's (http://rdp.cme.msu.edu/index.jsp) 16s database v16 or in the case of ITS1F, the RDP ITS V2 database. Operational Taxonomic Units (OTUs) contributing less than 1% of the total data have been excluded Report generation command used : $create_vsearch_single_sample_pdf_report_illumina.py KT-FF7_S6.merged.filt.otu.table.tsv S6 FF7 230508 16s

Taxanomical Classification

Kingdom Classification

Kingdom	Read Count	%
Bacteria	61605.0	99.64
Archaea	136.0	0.22
Unknown	87.0	0.14

Top Kingdom Classification

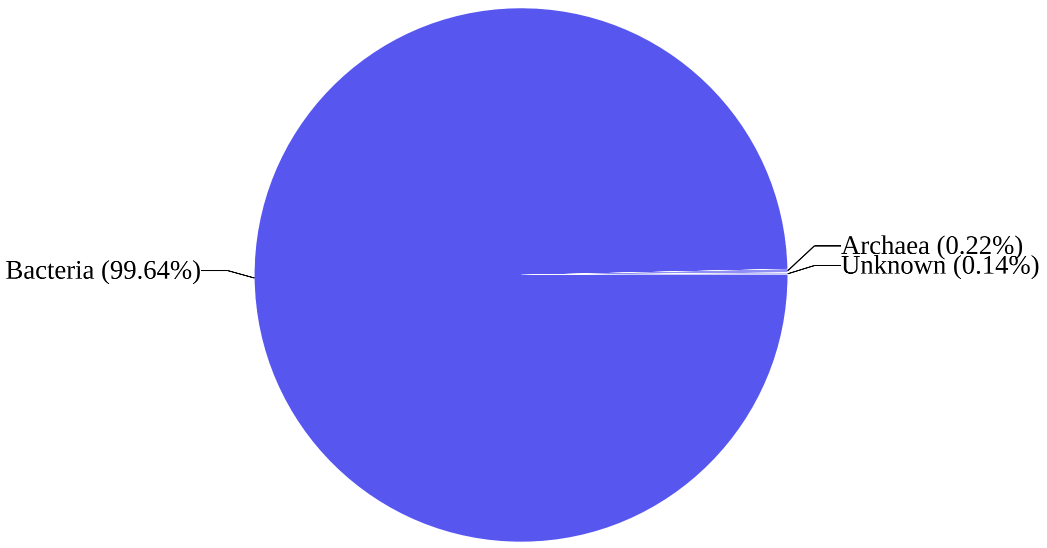

Phylum Classification

Phyla Classification	Read Count	%
Proteobacteria	37355.0	60.42
Actinobacteria	5468.0	8.84
Unknown	5240.0	8.48
Firmicutes	4118.0	6.66
Planctomycetes	2753.0	4.45
Bacteroidetes	1485.0	2.40
Cyanobacteria	1415.0	2.29
SBR1093	1322.0	2.14
Verrucomicrobia	805.0	1.30
WWE1	491.0	0.79
Chloroflexi	354.0	0.57
Spirochaetes	293.0	0.47
TM7	140.0	0.23
GN02	137.0	0.22
Acidobacteria	136.0	0.22
Euryarchaeota	101.0	0.16
Nitrospirae	55.0	0.09
WS2	40.0	0.06
Crenarchaeota	35.0	0.06
Synergistetes	35.0	0.06
WS3	23.0	0.04
Chlorobi	9.0	0.01
	9.0	0.01
Gemmatimonadetes	7.0	0.01

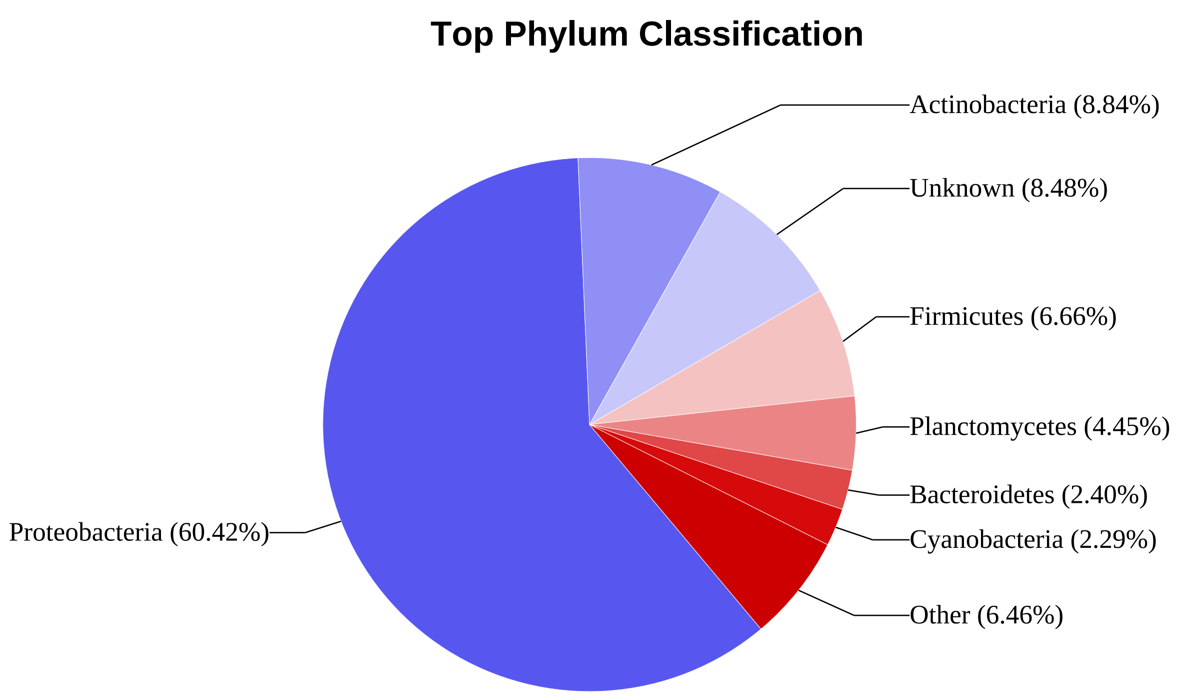
Top Phylum Classification
Actinobacteria (8.84%)
Unknown (8.48%)
Firmicutes (6.66%)
Planctomycetes (4.45%)
Bacteroidetes (2.40%)
Cyanobacteria (2.29%)
Other (6.46%)
Proteobacteria (60.42%)

Class Classification

Class	Read Count	%
Alphaproteobacteria	27207.0	44.01
Gammaproteobacteria	7545.0	12.20
Unknown	6921.0	11.19
Actinobacteria	4700.0	7.60
Bacilli	3649.0	5.90
Planctomycetia	2580.0	4.17
Betaproteobacteria	1247.0	2.02
Flavobacteriia	912.0	1.48
Deltaproteobacteria	884.0	1.43
Verrucomicrobiae	770.0	1.25
EC214	714.0	1.15
	654.0	1.06
VHS	604.0	0.98
Clostridia	469.0	0.76
Thermoleophilia	443.0	0.72
Spirochaetes	293.0	0.47
Cytophagia	207.0	0.33
Acidimicrobiia	183.0	0.30
Sphingobacteriia	158.0	0.26
Nostocophycideae	156.0	0.25
Anaerolineae	148.0	0.24
TM7	139.0	0.22
3BR	137.0	0.22
Acidobacteria	135.0	0.22
C6	133.0	0.22
Rubrobacteria	123.0	0.20
Thermomicrobia	119.0	0.19
Halobacteria	101.0	0.16
Ellin6529	87.0	0.14
Bacteroidia	81.0	0.13
Nitrospira	55.0	0.09
Synechococcophycideae	53.0	0.09
SHA	40.0	0.06
OM190	39.0	0.06
Thaumarchaeota	35.0	0.06
Synergistia	35.0	0.06
Oscillatoriophycideae	32.0	0.05
PRR	23.0	0.04
SJA	9.0	0.01

Gemm	7.0	0.01

Top Class Classification

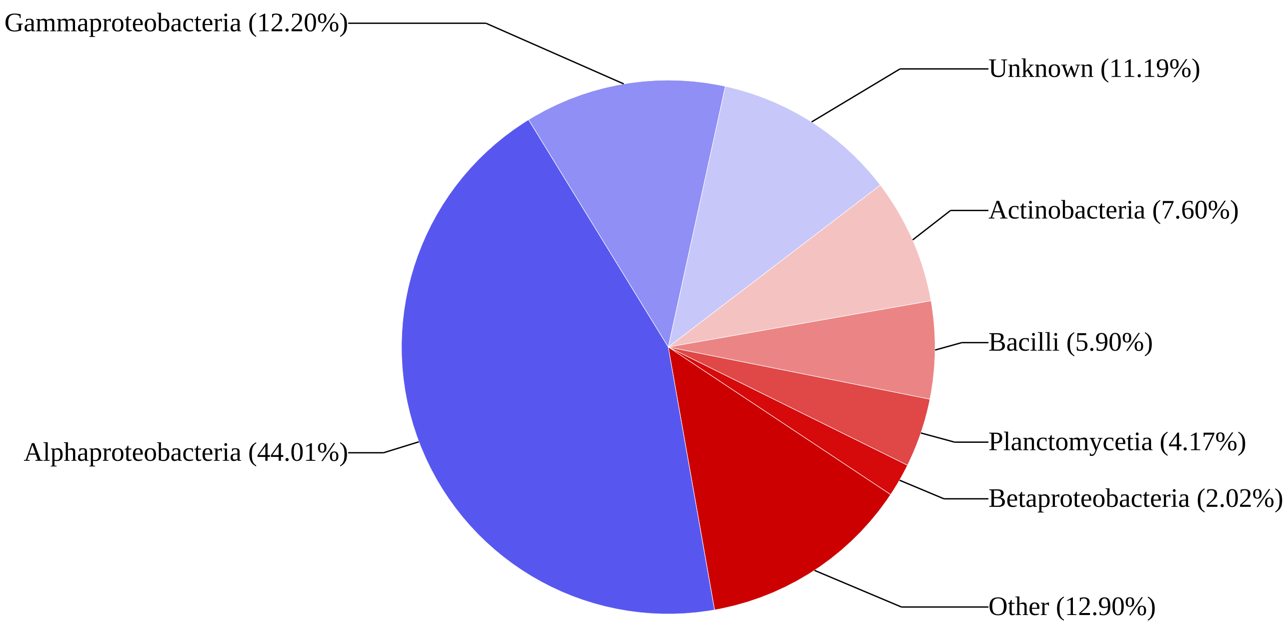

Order Classification

Order	Read Count	%
Unknown	16297.0	26.37
Rhizobiales	8270.0	13.38
Actinomycetales	4700.0	7.60
Sphingomonadales	4325.0	7.00
Rhodobacterales	4182.0	6.77
	3565.0	5.77
Oceanospirillales	3427.0	5.54
Bacillales	3048.0	4.93
Pirellulales	2147.0	3.47
Pseudomonadales	2129.0	3.44
Flavobacteriales	912.0	1.48
Enterobacteriales	791.0	1.28
Verrucomicrobiales	770.0	1.25
Burkholderiales	612.0	0.99
Methylophilales	609.0	0.99
Lactobacillales	579.0	0.94
Clostridiales	469.0	0.76
Spirobacillales	439.0	0.71
Gaiellales	415.0	0.67
Gemmatales	356.0	0.58
Alteromonadales	328.0	0.53
Caulobacterales	319.0	0.52
Rhodospirillales	255.0	0.41
NB1	237.0	0.38
Cytophagales	207.0	0.33
Acidimicrobiales	183.0	0.30
Sphingobacteriales	158.0	0.26
Nostocales	156.0	0.25
d113	133.0	0.22
Chromatiales	132.0	0.21
Rubrobacterales	123.0	0.20
JG30	118.0	0.19
BPC015	113.0	0.18
Thiotrichales	106.0	0.17
Halobacteriales	101.0	0.16
Spirochaetales	93.0	0.15
GMD14H09	83.0	0.13
Bacteroidales	81.0	0.13
Vibrionales	78.0	0.13

Aeromonadales	71.0	0.11
Planctomycetales	71.0	0.11
SHA	69.0	0.11
Caldilineales	62.0	0.10
Kiloniellales	57.0	0.09
Nitrospirales	55.0	0.09
Synechococcales	52.0	0.08
Legionellales	45.0	0.07
agg27	39.0	0.06
Synergistales	35.0	0.06
Nitrososphaerales	33.0	0.05
Myxococcales	32.0	0.05
Solirubrobacterales	28.0	0.05
GN03	23.0	0.04
iii1	22.0	0.04
Salinisphaerales	13.0	0.02
Thiohalorhabdales	13.0	0.02
SBR1031	12.0	0.02
Syntrophobacterales	12.0	0.02
Desulfobacterales	12.0	0.02
PYR10d3	8.0	0.01

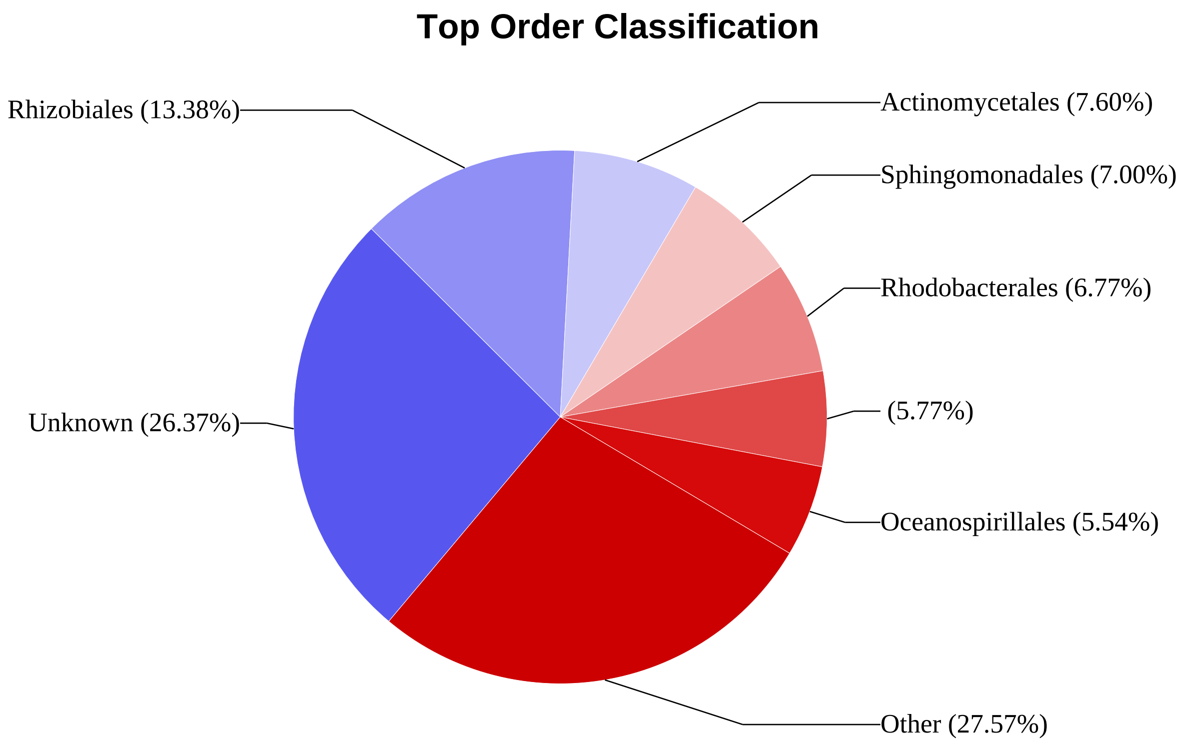

Family Classification

Family	Read Count	%
Unknown	24459.0	39.57
Hyphomicrobiaceae	6276.0	10.15
Rhodobacteraceae	4128.0	6.68
Sphingomonadaceae	3798.0	6.14
Endozoicimonaceae	3349.0	5.42
Pirellulaceae	2147.0	3.47
Moraxellaceae	1681.0	2.72
Methylobacteriaceae	1451.0	2.35
Bacillaceae	1263.0	2.04
Mycobacteriaceae	901.0	1.46
Enterobacteriaceae	791.0	1.28
Verrucomicrobiaceae	770.0	1.25
Propionibacteriaceae	764.0	1.24
Flavobacteriaceae	656.0	1.06
Methylophilaceae	609.0	0.99
Erythrobacteraceae	523.0	0.85
Dietziaceae	512.0	0.83
Staphylococcaceae	461.0	0.75
Pseudomonadaceae	448.0	0.72
Corynebacteriaceae	442.0	0.72
Streptomycetaceae	387.0	0.63
Comamonadaceae	387.0	0.63
Caulobacteraceae	319.0	0.52
Micrococcaceae	288.0	0.47
Brevibacteriaceae	252.0	0.41
Oxalobacteraceae	225.0	0.36
Ruminococcaceae	208.0	0.34
Streptococcaceae	204.0	0.33
Lactobacillaceae	204.0	0.33
Acetobacteraceae	166.0	0.27
Nocardioidaceae	165.0	0.27
OM60	161.0	0.26
Sphingobacteriaceae	158.0	0.26
Microbacteriaceae	148.0	0.24
Enterococcaceae	147.0	0.24
Isosphaeraceae	137.0	0.22
Rubrobacteraceae	123.0	0.20
Nostocaceae	114.0	0.18
Planococcaceae	110.0	0.18

Piscirickettsiaceae	106.0	0.17
Dermabacteraceae	103.0	0.17
Halobacteriaceae	101.0	0.16
Peptostreptococcaceae	101.0	0.16
Phyllobacteriaceae	97.0	0.16
Gemmataceae	95.0	0.15
Spirochaetaceae	93.0	0.15
Paenibacillaceae	91.0	0.15
Alteromonadaceae	89.0	0.14
Nocardiaceae	84.0	0.14
Halomonadaceae	78.0	0.13
Aeromonadaceae	71.0	0.11
Planctomycetaceae	71.0	0.11
Bacteroidaceae	71.0	0.11
Cryomorphaceae	69.0	0.11
Chitinophagaceae	68.0	0.11
Brucellaceae	67.0	0.11
Flammeovirgaceae	64.0	0.10
Caldilineaceae	62.0	0.10
Nitrospiraceae	55.0	0.09
Hyphomonadaceae	54.0	0.09
Clostridiaceae	54.0	0.09
Intrasporangiaceae	52.0	0.08
Rhodospirillaceae	49.0	0.08
Gordoniaceae	42.0	0.07
Xanthobacteraceae	42.0	0.07
Vibrionaceae	39.0	0.06
Synechococcaceae	35.0	0.06
koll13	35.0	0.06
TTA_B6	34.0	0.06
Nitrososphaeraceae	33.0	0.05
Cohaesibacteraceae	31.0	0.05
Pseudoalteromonadaceae	31.0	0.05
Gaiellaceae	27.0	0.04
Rhizobiaceae	27.0	0.04
Saprospiraceae	25.0	0.04
Rhodothermaceae	24.0	0.04
Micromonosporaceae	21.0	0.03
Methylocystaceae	19.0	0.03
Cytophagaceae	19.0	0.03
JdFBGBact	18.0	0.03
Listeriaceae	17.0	0.03
Acaryochloridaceae	17.0	0.03

Salinisphaeraceae	13.0	0.02
Cystobacterineae	13.0	0.02
A4b	12.0	0.02
Syntrophobacteraceae	12.0	0.02
Desulfobulbaceae	11.0	0.02
Rikenellaceae	10.0	0.02
Lachnospiraceae	10.0	0.02
Pseudonocardiaceae	8.0	0.01
Dermacoccaceae	7.0	0.01

Top Family Classification

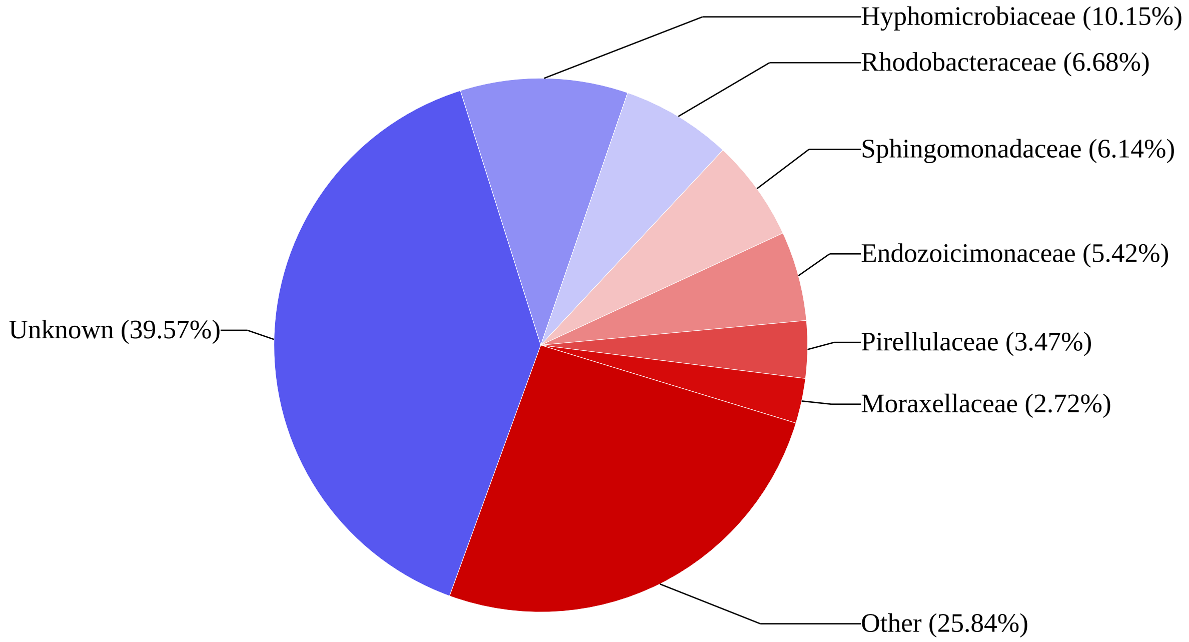

Genus Classification

Genus	Read Count	%
Unknown	43235.0	69.99
Sphingomonas	3545.0	5.74
Methylobacterium	1418.0	2.30
Psychrobacter	1250.0	2.02
Paracoccus	1079.0	1.75
Bacillus	999.0	1.62
Mycobacterium	901.0	1.46
Propionibacterium	761.0	1.23
Staphylococcus	485.0	0.79
Corynebacterium	442.0	0.72
Dietzia	430.0	0.70
planctomycete	389.0	0.63
Acinetobacter	384.0	0.62
Streptomyces	375.0	0.61
Endozoicomonas	343.0	0.56
Brevibacterium	252.0	0.41
Pseudozobellia	226.0	0.37
Massilia	218.0	0.35
Streptococcus	196.0	0.32
Nitrobacteria	196.0	0.32
Lactobacillus	196.0	0.32
Exiguobacterium	190.0	0.31
Pseudomonas	179.0	0.29
Hyphomicrobium	167.0	0.27
Planctomycete	167.0	0.27
Sphingobacterium	156.0	0.25
Persicirhabdus	154.0	0.25
Coccinimonas	151.0	0.24
Enterococcus	146.0	0.24
Cloacibacterium	135.0	0.22
Rubrobacter	123.0	0.20
Clostridium	118.0	0.19
Oceanobacillus	101.0	0.16
Kocuria	101.0	0.16
Ucs1325	101.0	0.16
Novosphingobium	99.0	0.16
Treponema	92.0	0.15
Micrococcus	84.0	0.14
Brachybacterium	84.0	0.14
Caulobacter	82.0	0.13
Paenibacillus	81.0	0.13

Rhodococcus	81.0	0.13
Hoeflea	81.0	0.13
Halomonas	78.0	0.13
Nocardioides	74.0	0.12
Aeromonas	71.0	0.11
Planctomyces	71.0	0.11
Bacteroides	71.0	0.11
Haliea	70.0	0.11
Salipiger	66.0	0.11
Halococcus	62.0	0.10
Ochrobactrum	58.0	0.09
Craurococcus	57.0	0.09
Hyphomonas	53.0	0.09
Oscillospira	53.0	0.09
Erythrobacter	51.0	0.08
Gemmata	49.0	0.08
Chryseobacterium	44.0	0.07
Friedmanniella	43.0	0.07
Marmoricola	43.0	0.07
Gordonia	42.0	0.07
Arenibacter	41.0	0.07
Brevundimonas	39.0	0.06
Microbispora	38.0	0.06
mixed	35.0	0.06
E6	34.0	0.06
BD2	31.0	0.05
Ruminococcus	28.0	0.05
Kytococcus	27.0	0.04
Alishewanella	27.0	0.04
Enhydrobacter	26.0	0.04
Candidatus	26.0	0.04
Arthrobacter	26.0	0.04
Ruegeria	26.0	0.04
Halalkalicoccus	25.0	0.04
Rubricoccus	24.0	0.04
Sediminibacterium	22.0	0.04
Prochlorococcus	21.0	0.03
Methylopila	19.0	0.03
Hymenobacter	19.0	0.03
Acaryochloris	17.0	0.03
Marihabitans	16.0	0.03
Cohaesibacter	15.0	0.02
Filomicrobium	14.0	0.02

Brochothrix	14.0	0.02
Salinisphaera	13.0	0.02
Alistipes	10.0	0.02
Brevibacillus	10.0	0.02
Rhizobium	9.0	0.01
Epulopiscium	9.0	0.01
Verrucomicrobium	9.0	0.01
Roseovarius	9.0	0.01
Methylotenera	8.0	0.01
Lactococcus	8.0	0.01
Synechococcus	8.0	0.01
Vibrio	7.0	0.01
Pseudonocardia	7.0	0.01
Dermacoccus	7.0	0.01

Top Genus Classification

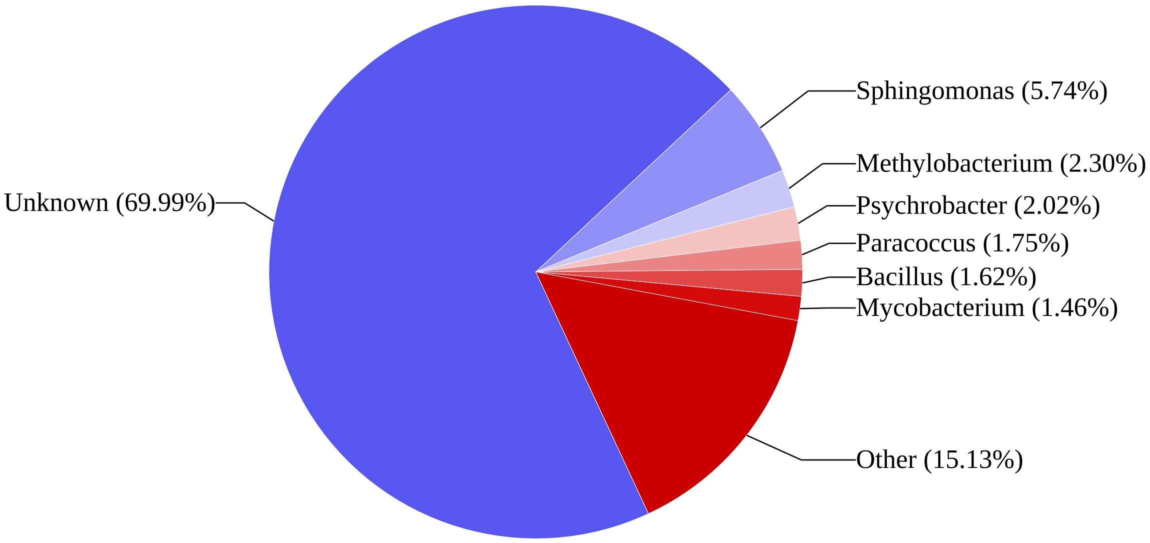

Species Classification

Species	Read Count	%
Unknown	40654.0	65.85
Unknown_	13363.0	21.65
Paracoccus_zeaxanthinifaciens	988.0	1.60
Psychrobacter_celer	576.0	0.93
planctomycete_MS1399	266.0	0.43
Corynebacterium_	226.0	0.37
Pseudozobellia_thermophila	226.0	0.37
Psychrobacter_meningitidis	207.0	0.34
Psychrobacter_pacificensis	200.0	0.32
Nitrobacteria_hamadaniensis	196.0	0.32
Propionibacterium_acnes	194.0	0.31
Exiguobacterium_	190.0	0.31
Planctomycete_LF1	167.0	0.27
Hyphomicrobium_	158.0	0.26
Persicirhabdus_	154.0	0.25
Coccinimonas_marina	151.0	0.24
Lactobacillus_iners	147.0	0.24
Sphingobacterium_	143.0	0.23
Cloacibacterium_	135.0	0.22
Rubrobacter_	123.0	0.20
Acinetobacter_rhizosphaerae	121.0	0.20
Propionibacterium_granulosum	119.0	0.19
Psychrobacter_marincola	119.0	0.19
Mycobacterium_vaccae	109.0	0.18
Kocuria_palustris	101.0	0.16
Ucs1325_	101.0	0.16
Acinetobacter_guillouiae	94.0	0.15
Treponema_	92.0	0.15
Clostridium_bartlettii	91.0	0.15
Oceanobacillus_	91.0	0.15
Paracoccus_marcusii	90.0	0.15
Brachybacterium_	84.0	0.14
Micrococcus_	83.0	0.13
Planctomyces_	71.0	0.12
Bacteroides_	71.0	0.12
Haliea_mediterranea	70.0	0.11
Salipiger_mucosus	66.0	0.11
Halococcus_	60.0	0.10
Craurococcus_roseus	57.0	0.09

Ochrobactrum_intermedium	55.0	0.09
Staphylococcus_sciuri	54.0	0.09
Psychrobacter_	54.0	0.09
Oscillospira_	53.0	0.09
Paenibacillus_	52.0	0.08
Brevibacterium_	52.0	0.08
Rhodococcus_	50.0	0.08
Gemmata_	49.0	0.08
Hyphomonas_	48.0	0.08
Chryseobacterium_	44.0	0.07
Friedmanniella_	43.0	0.07
Gordonia_	42.0	0.07
Marmoricola_aurantiacus	42.0	0.07
Arenibacter_	41.0	0.07
Brevundimonas_	39.0	0.06
Microbispora_rosea	38.0	0.06
Acinetobacter_lwoffii	37.0	0.06
Bacillus_firmus	37.0	0.06
mixed_culture	35.0	0.06
E6_	34.0	0.06
Pseudomonas_stutzeri	34.0	0.06
Novosphingobium_capsulatum	33.0	0.05
BD2_	31.0	0.05
Acinetobacter_venetianus	31.0	0.05
Ruminococcus_	28.0	0.05
Kytococcus_	27.0	0.04
Alishewanella_	27.0	0.04
Brevibacterium_casei	26.0	0.04
Enhydrobacter_aerosaccus	26.0	0.04
Candidatus_	26.0	0.04
Ruegeria_lacuscaerulensis	26.0	0.04
Halalkalicoccus_tibetensis	25.0	0.04
Rubricoccus_	24.0	0.04
Staphylococcus_saprophyticus	24.0	0.04
Sediminibacterium_	22.0	0.04
Psychrobacter_pulmonis	21.0	0.03
Prochlorococcus_	21.0	0.03
Methylopila_	19.0	0.03
Hymenobacter_	19.0	0.03
Paenibacillus_lautus	18.0	0.03
Acaryochloris_	17.0	0.03
Sphingomonas_azotifigens	16.0	0.03
Pseudomonas_balearica	16.0	0.03

Marihabitans_asiaticum	16.0	0.03
Salinisphaera_	13.0	0.02
Alistipes_onderdonkii	9.0	0.01
Epulopiscium_	9.0	0.01
Verrucomicrobium_	9.0	0.01
Roseovarius_	9.0	0.01
Methylotenera_versatilis	8.0	0.01
Lactococcus_garvieae	8.0	0.01
Synechococcus_	8.0	0.01
Methylobacterium_mesophilicum	7.0	0.01
Pseudonocardia_halophobica	7.0	0.01
Dermacoccus_	7.0	0.01
Caulobacter_vibrioides	7.0	0.01
Brochothrix_	7.0	0.01

Top Species Classification

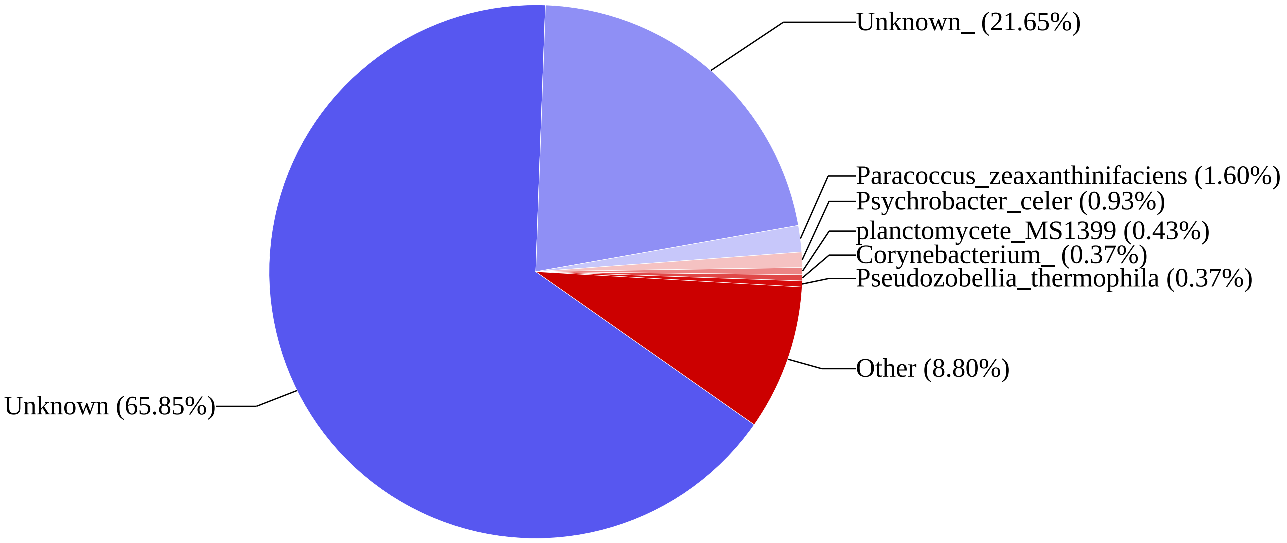

---------- End of report ----------
