# Supplementary material for: Metabolic Profiling, Antiviral Activity and the Microbiome of Some Mauritian Soft Corals
Source: Mar Drugs. 2023 Oct 31;21(11):574. doi: 10.3390/md21110574 (PMC10672535; doi:10.3390/md21110574)
Supplement: Supplementary file 1 [file marinedrugs-21-00574-s001.zip › Supplementary data S4- S.polydactyla.pdf]

inqaba biotec™

Africa's Genomics Company

inqaba biotec metagenomic report

Sample Information

Index:	S3
Sample Name:	SF2
Run Name:	230508
Report Date:	Thu May 11 08:40:49 2023

This report contains the summarized metagenomic analysis of 16s/ITS1F gene sequencing. Samples were sequenced on an illumina system (www.illumina.com). Reads were processed through usearch (https://drive5.com/usearch) and taxonomic information was determined based on the Ribosomal Database Project's (http://rdp.cme.msu.edu/index.jsp) 16s database v16 or in the case of ITS1F, the RDP ITS V2 database. Operational Taxonomic Units (OTUs) contributing less than 1% of the total data have been excluded Report generation command used :
$create_vsearch_single_sample_pdf_report_illumina.py KT-SF2_S3.merged.filt.otu.table.tsv S3 SF2 230508 16s

Taxanomical Classification

Kingdom Classification

Kingdom	Read Count	%
Bacteria	77959.0	99.97
Archaea	26.0	0.03

Top Kingdom Classification

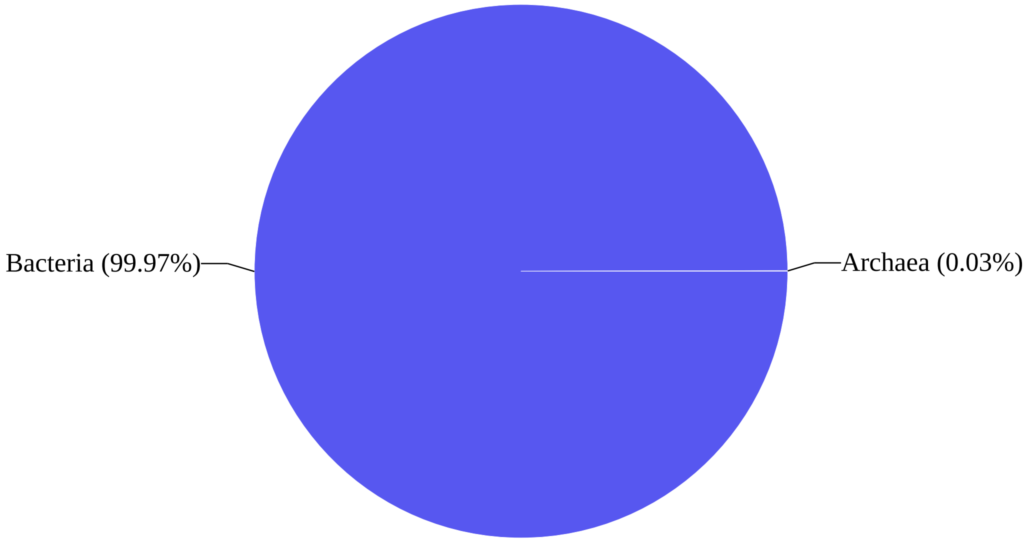

Phylum Classification

Phyla Classification	Read Count	%
Unknown	35484.0	45.51
Spirochaetes	18456.0	23.67
Proteobacteria	14683.0	18.83
Firmicutes	4049.0	5.19
Planctomycetes	1546.0	1.98
Actinobacteria	1539.0	1.97
Bacteroidetes	776.0	1.00
Cyanobacteria	708.0	0.91
Verrucomicrobia	235.0	0.30
Chloroflexi	207.0	0.27
Acidobacteria	69.0	0.09
Lentisphaerae	51.0	0.07
Fusobacteria	33.0	0.04
SBR1093	28.0	0.04
Euryarchaeota	25.0	0.03
	24.0	0.03
WWE1	22.0	0.03
TM7	18.0	0.02
Gemmatimonadetes	11.0	0.01

Top Phylum Classification

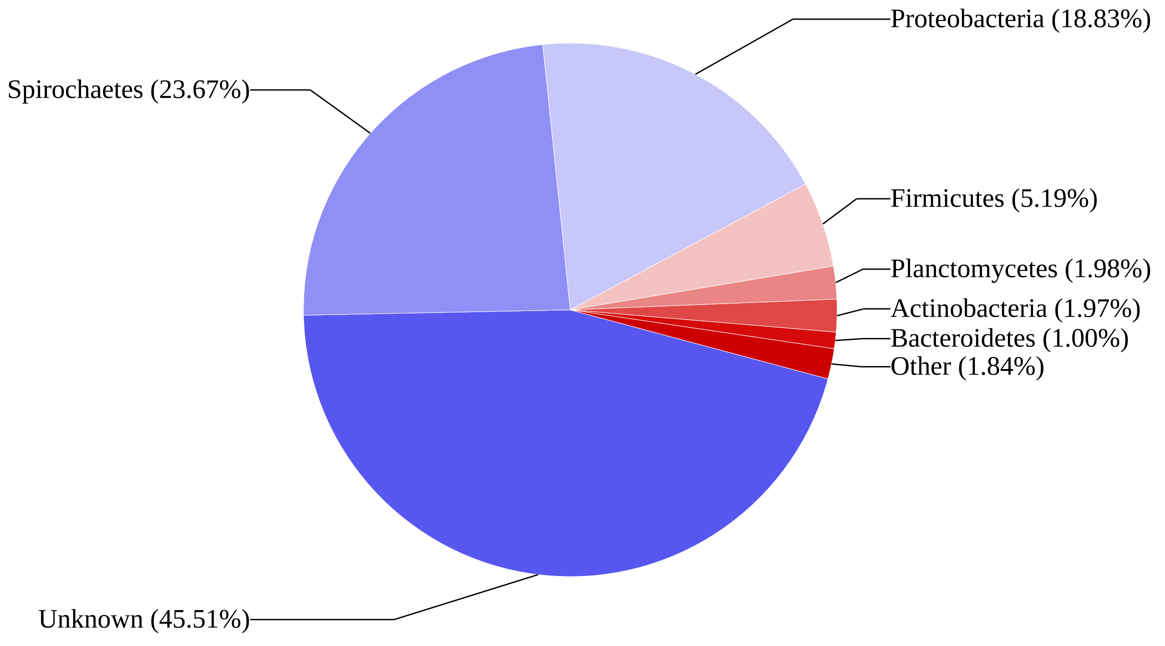

Class Classification

Class	Read Count	%
Unknown	35767.0	45.89
Spirochaetes	18456.0	23.68
Gammaproteobacteria	12600.0	16.17
Bacilli	3395.0	4.36
Alphaproteobacteria	1727.0	2.22
Actinobacteria	1201.0	1.54
Planctomycetia	1094.0	1.40
Clostridia	643.0	0.82
Flavobacteriia	384.0	0.49
	356.0	0.46
Oscillatoriophycideae	346.0	0.44
OM190	223.0	0.29
Deltaproteobacteria	218.0	0.28
Phycisphaerae	188.0	0.24
Verrucomicrobiae	183.0	0.23
Acidimicrobiia	175.0	0.22
Synechococcophycideae	148.0	0.19
Thermomicrobia	122.0	0.16
Bacteroidia	108.0	0.14
Anaerolineae	83.0	0.11
Betaproteobacteria	69.0	0.09
Thermoleophilia	66.0	0.08
Coriobacteriia	57.0	0.07
Cytophagia	48.0	0.06
Opitutae	42.0	0.05
Fusobacteriia	33.0	0.04
Sva0725	26.0	0.03
Gloeobacterophycideae	24.0	0.03
Halobacteria	22.0	0.03
Acidobacteria	19.0	0.02
EC214	19.0	0.02
TM7	18.0	0.02
Rubrobacteria	16.0	0.02
Gemmatimonadetes	11.0	0.01
vadinHA49	11.0	0.01
TA18	10.0	0.01
BME43	10.0	0.01
VHS	9.0	0.01
Pla3	9.0	0.01

Solibacteres	8.0	0.01

Top Class Classification

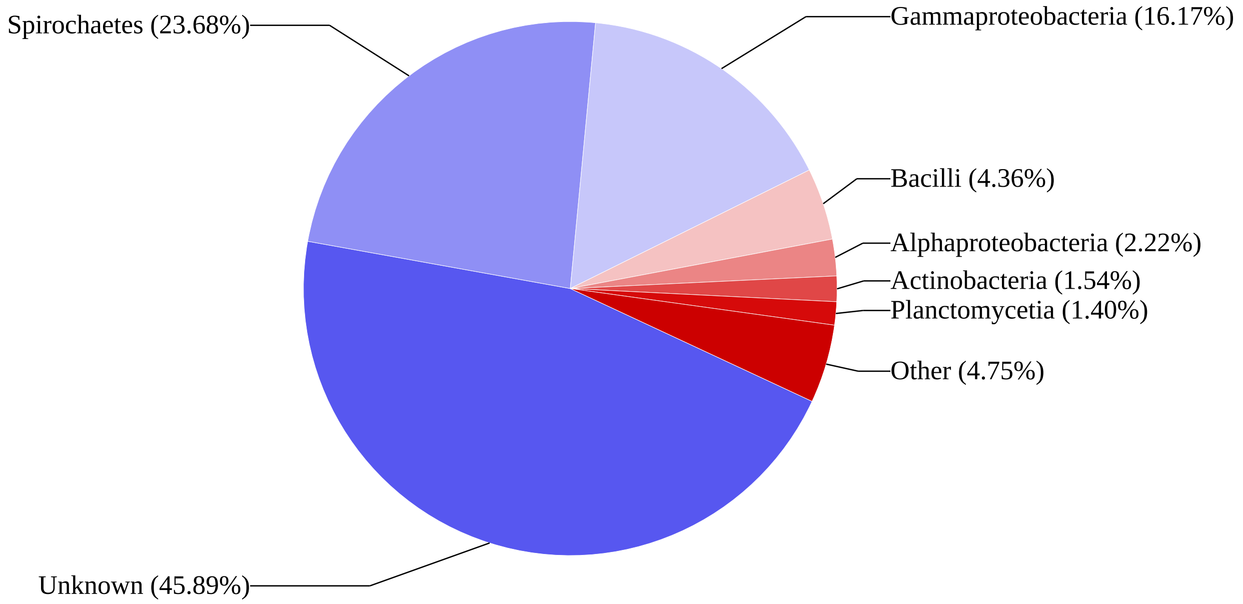

Order Classification

Order	Read Count	%
Unknown	36645.0	47.04
Spirochaetales	18449.0	23.68
Oceanospirillales	11459.0	14.71
Lactobacillales	3137.0	4.03
Actinomycetales	1168.0	1.50
Pirellulales	1039.0	1.33
Clostridiales	638.0	0.82
Rhodobacterales	557.0	0.72
	408.0	0.52
Flavobacteriales	384.0	0.49
Rhodospirillales	360.0	0.46
Sphingomonadales	264.0	0.34
Oscillatoriales	232.0	0.30
Rhizobiales	232.0	0.30
Alteromonadales	192.0	0.25
Verrucomicrobiales	183.0	0.23
Acidimicrobiales	175.0	0.22
Phycisphaerales	174.0	0.22
Bacillales	163.0	0.21
CL500	149.0	0.19
Pseudomonadales	128.0	0.16
Chroococcales	111.0	0.14
Bacteroidales	108.0	0.14
Thiotrichales	105.0	0.13
Myxococcales	105.0	0.13
Pseudanabaenales	96.0	0.12
Enterobacteriales	91.0	0.12
agg27	74.0	0.09
Coriobacteriales	57.0	0.07
Vibrionales	56.0	0.07
Z20	51.0	0.07
Cytophagales	48.0	0.06
Gaiellales	47.0	0.06
Planctomycetales	46.0	0.06
Xanthomonadales	44.0	0.06
Kiloniellales	43.0	0.06
Synechococcales	38.0	0.05
Caldilineales	38.0	0.05
NB1	36.0	0.05

SBR1031	35.0	0.04
Burkholderiales	35.0	0.04
Fusobacteriales	33.0	0.04
Puniceicoccales	32.0	0.04
Bifidobacteriales	31.0	0.04
HOC36	27.0	0.03
Sva0725	26.0	0.03
Gloeobacterales	24.0	0.03
Methylophilales	22.0	0.03
Halobacteriales	22.0	0.03
Thiohalorhabdales	20.0	0.03
BD7	19.0	0.02
HTCC2188	19.0	0.02
Desulfobacterales	19.0	0.02
Solirubrobacterales	19.0	0.02
Rubrobacterales	16.0	0.02
JG30	14.0	0.02
Chromatiales	14.0	0.02
RB41	13.0	0.02
iii1	12.0	0.02
Kordiimonadales	11.0	0.01
Gemmatimonadales	11.0	0.01
Bdellovibrionales	11.0	0.01
Salinisphaerales	11.0	0.01
DH61	11.0	0.01
CCM11a	10.0	0.01
Caulobacterales	9.0	0.01
I025	9.0	0.01
Spirobacillales	9.0	0.01
Neisseriales	8.0	0.01
Solibacterales	8.0	0.01
Gemmatales	8.0	0.01

Top Order Classification

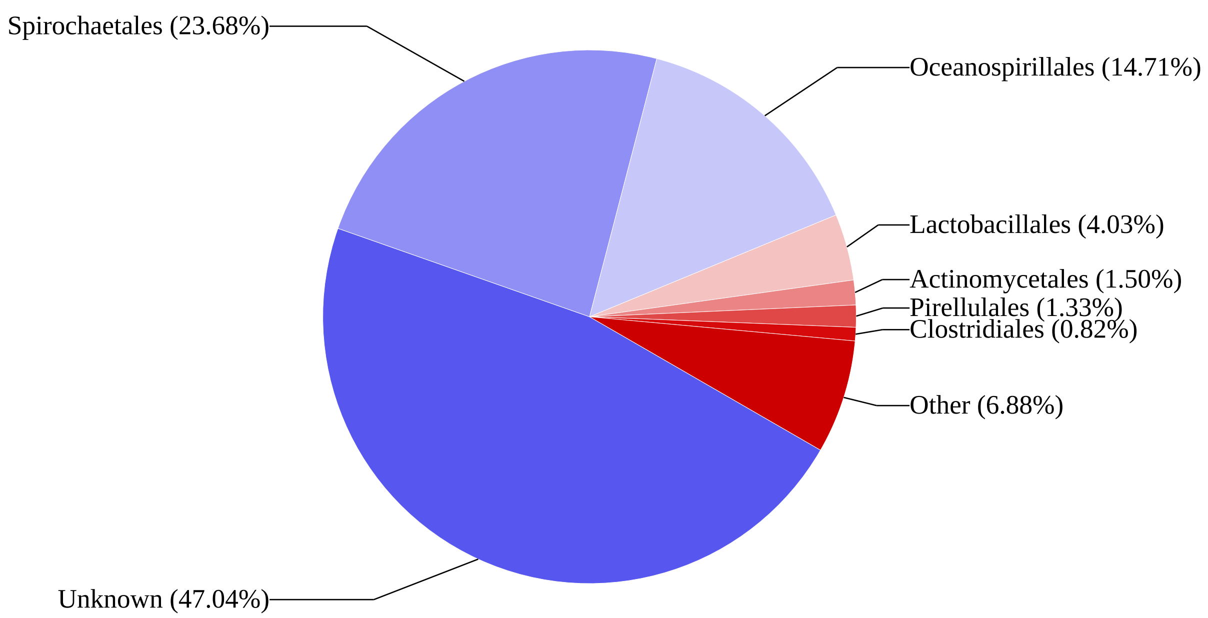

Family Classification

Family	Read Count	%
Unknown	38852.0	49.90
Spirochaetaceae	18449.0	23.70
Endozoicimonaceae	11454.0	14.71
Lactobacillaceae	1769.0	2.27
Pirellulaceae	1039.0	1.33
Streptococcaceae	680.0	0.87
Rhodobacteraceae	530.0	0.68
Flavobacteriaceae	356.0	0.46
Ruminococcaceae	347.0	0.45
Acetobacteraceae	320.0	0.41
Sphingomonadaceae	236.0	0.30
Phormidiaceae	232.0	0.30
Actinosynnemataceae	204.0	0.26
Verrucomicrobiaceae	183.0	0.24
Methylobacteriaceae	174.0	0.22
Saprospiraceae	143.0	0.18
Corynebacteriaceae	125.0	0.16
Piscirickettsiaceae	105.0	0.13
Pseudanabaenaceae	96.0	0.12
Propionibacteriaceae	96.0	0.12
OM60	96.0	0.12
Actinomycetaceae	95.0	0.12
Enterobacteriaceae	91.0	0.12
Lachnospiraceae	91.0	0.12
Micrococcaceae	76.0	0.10
Bacillaceae	72.0	0.09
Leuconostocaceae	70.0	0.09
Pseudomonadaceae	70.0	0.09
Xenococcaceae	65.0	0.08
C111	58.0	0.07
Moraxellaceae	58.0	0.07
Coriobacteriaceae	57.0	0.07
Phycisphaeraceae	53.0	0.07
Microbacteriaceae	52.0	0.07
Alteromonadaceae	52.0	0.07
Nocardioidaceae	51.0	0.07
R4	51.0	0.07
wb1_P06	50.0	0.06
Enterococcaceae	48.0	0.06

Vibrionaceae	47.0	0.06
Planctomycetaceae	46.0	0.06
Clostridiaceae	44.0	0.06
Kineosporiaceae	44.0	0.06
Xanthomonadaceae	44.0	0.06
Gaiellaceae	41.0	0.05
Acaryochloridaceae	38.0	0.05
Caldilineaceae	38.0	0.05
A4b	35.0	0.04
Comamonadaceae	35.0	0.04
Prevotellaceae	34.0	0.04
Veillonellaceae	34.0	0.04
Fusobacteriaceae	33.0	0.04
Puniceicoccaceae	32.0	0.04
Bifidobacteriaceae	31.0	0.04
Staphylococcaceae	31.0	0.04
Haliangiaceae	30.0	0.04
JdFBGBact	24.0	0.03
Gloeobacteraceae	24.0	0.03
Phyllobacteriaceae	23.0	0.03
Rhodospirillaceae	22.0	0.03
Methylophilaceae	22.0	0.03
Halobacteriaceae	22.0	0.03
Streptomycetaceae	22.0	0.03
Hyphomonadaceae	20.0	0.03
Bacteroidaceae	20.0	0.03
Cryomorphaceae	20.0	0.03
Desulfobulbaceae	19.0	0.02
OM27	19.0	0.02
Intrasporangiaceae	19.0	0.02
Flammeovirgaceae	18.0	0.02
Geodermatophilaceae	17.0	0.02
JTB38	16.0	0.02
Pseudonocardiaceae	16.0	0.02
Rubrobacteraceae	16.0	0.02
Brevibacteriaceae	15.0	0.02
HTCC2089	15.0	0.02
Erythrobacteraceae	15.0	0.02
Dermabacteraceae	13.0	0.02
Ellin6075	13.0	0.02
Peptostreptococcaceae	13.0	0.02
Carnobacteriaceae	13.0	0.02
Hyphomicrobiaceae	12.0	0.02

Cytophagaceae	12.0	0.02
Bacteriovoracaceae	11.0	0.01
Salinisphaeraceae	11.0	0.01
Nannocystaceae	10.0	0.01
Caulobacteraceae	9.0	0.01
Paenibacillaceae	9.0	0.01
Cyanobacteriaceae	9.0	0.01
Porphyromonadaceae	9.0	0.01
mb2424	9.0	0.01
Neisseriaceae	8.0	0.01
Gemmataceae	8.0	0.01

Top Family Classification

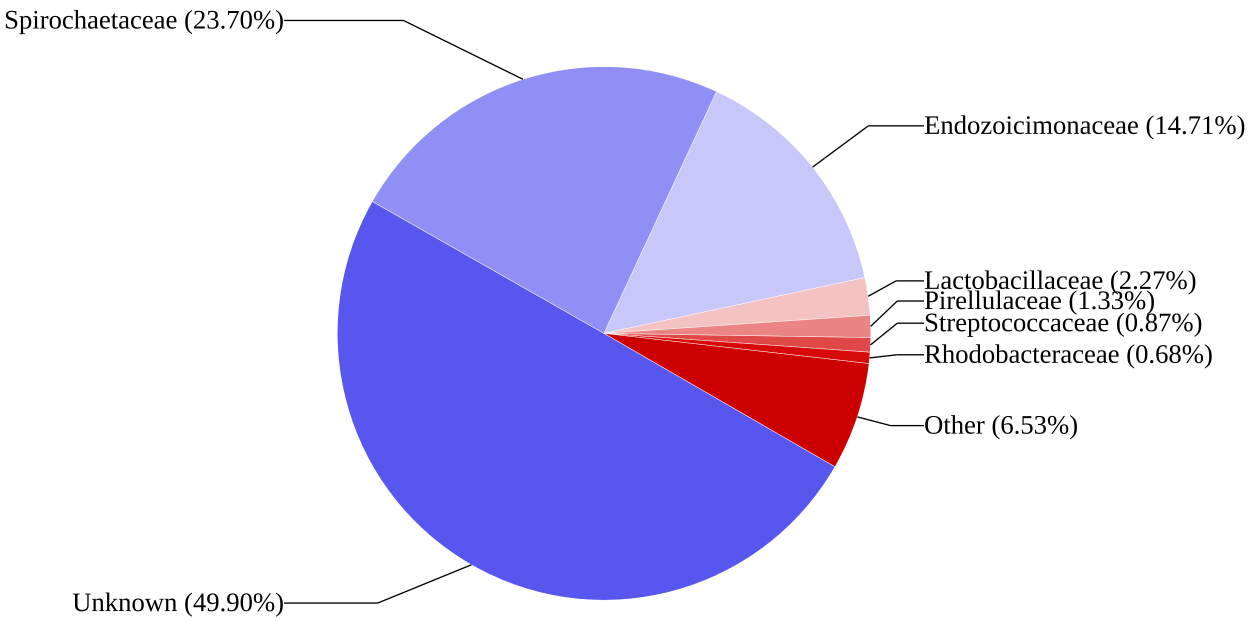

Genus Classification

Genus	Read Count	%
Unknown	52744.0	67.87
Spirochaeta	18443.0	23.73
Endozoicomonas	1763.0	2.27
Lactobacillus	1207.0	1.55
Streptococcus	665.0	0.86
Acetobacter	221.0	0.28
Sphingomonas	204.0	0.26
Methylobacterium	174.0	0.22
Lyngbya	166.0	0.21
Ruminococcus	130.0	0.17
Corynebacterium	125.0	0.16
Ethanoligenens	96.0	0.12
Propionibacterium	94.0	0.12
Planctomycete	93.0	0.12
planctomycete	89.0	0.11
Ruegeria	64.0	0.08
Muricauda	62.0	0.08
Weissella	56.0	0.07
Micrococcus	55.0	0.07
Ascidianibacter	48.0	0.06
Rubritalea	48.0	0.06
Planctomyces	46.0	0.06
Pediococcus	44.0	0.06
Clostridium	44.0	0.06
Haliea	43.0	0.06
Enterococcus	43.0	0.06
Acinetobacter	41.0	0.05
Nocardioides	35.0	0.05
Xenococcus	35.0	0.05
Prevotella	34.0	0.04
Staphylococcus	33.0	0.04
Propionigenium	32.0	0.04
Coraliomargarita	32.0	0.04
Kineococcus	31.0	0.04
Collinsella	29.0	0.04
Croceitalea	26.0	0.03
Congregibacter	26.0	0.03
Halomicronema	24.0	0.03
Gloeobacter	24.0	0.03
Halococcus	22.0	0.03
Pseudomonas	21.0	0.03

Acaryochloris	21.0	0.03
Hoeflea	19.0	0.02
Streptomyces	19.0	0.02
Haloferula	17.0	0.02
Bifidobacterium	16.0	0.02
Rubrobacter	16.0	0.02
Brevibacterium	15.0	0.02
Microbacterium	15.0	0.02
Psychrobacter	15.0	0.02
Xanthomonas	14.0	0.02
Ilumatobacter	13.0	0.02
Bacillus	13.0	0.02
Alteromonas	13.0	0.02
Shimia	13.0	0.02
Isobaculum	13.0	0.02
Coccinimonas	12.0	0.02
Bacteroides	12.0	0.02
Fluviicola	12.0	0.02
Geodermatophilus	12.0	0.02
Winogradskyella	12.0	0.02
Hydrocoleum	12.0	0.02
Stanieria	12.0	0.02
Succiniclasticum	12.0	0.02
Persicobacter	12.0	0.02
Skermanella	11.0	0.01
Salinisphaera	11.0	0.01
SC3	11.0	0.01
Adlercreutzia	11.0	0.01
Actinomycetospora	10.0	0.01
Psychroserpens	10.0	0.01
Lysobacter	10.0	0.01
Plesiocystis	10.0	0.01
Candidatus	10.0	0.01
Tenacibaculum	10.0	0.01
A17	9.0	0.01
Dysgonomonas	9.0	0.01
Rhodopirellula	9.0	0.01
Rubellimicrobium	9.0	0.01
Agreia	8.0	0.01
Marmoricola	8.0	0.01

Top Genus Classification

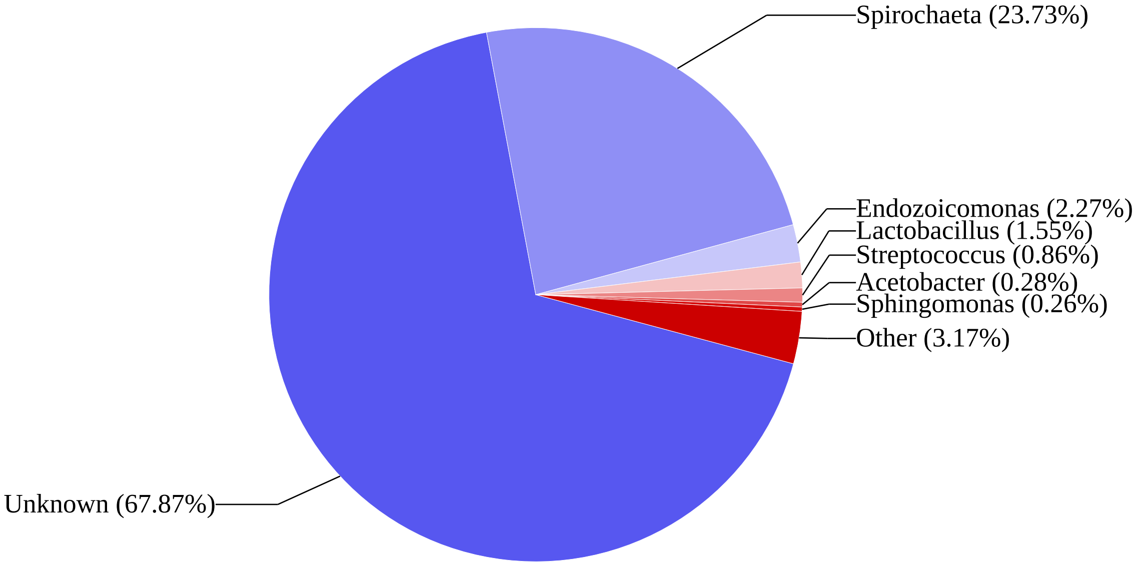
Spirochaeta (23.73%)
Endozoicomonas (2.27%)
Lactobacillus (1.55%)
Streptococcus (0.86%)
Acetobacter (0.28%)
Sphingomonas (0.26%)
Other (3.17%)
Unknown (67.87%)

Species Classification

Species	Read Count	%
Unknown	54619.0	70.33
Spirochaeta_	18384.0	23.67
Unknown_	2339.0	3.01
Lactobacillus_mucosae	725.0	0.93
Ruminococcus_	130.0	0.17
Lactobacillus_paraplantarum	113.0	0.15
Ethanoligenens_	96.0	0.12
Planctomycete_LF1	93.0	0.12
Corynebacterium_	65.0	0.08
Lactobacillus_manihotivorans	55.0	0.07
Ascidianibacter_aurantiacus	48.0	0.06
Rubritalea_	48.0	0.06
Planctomyces_	46.0	0.06
Propionibacterium_acnes	43.0	0.06
Haliea_mediterranea	43.0	0.06
planctomycete_MS1399	42.0	0.05
Muricauda_olearia	39.0	0.05
Xenococcus_	35.0	0.05
Micrococcus_	35.0	0.05
Prevotella_	33.0	0.04
Propionigenium_	32.0	0.04
Lactobacillus_	32.0	0.04
Coraliomargarita_	32.0	0.04
Streptococcus_equi	31.0	0.04
Kineococcus_	30.0	0.04
Congregibacter_	26.0	0.03
Collinsella_stercoris	26.0	0.03
Halomicronema_	24.0	0.03
Gloeobacter_	24.0	0.03
Acaryochloris_	21.0	0.03
Halococcus_	19.0	0.02
Croceitalea_dokdonensis	18.0	0.02
Clostridium_tyrobutyricum	17.0	0.02
Rubrobacter_	16.0	0.02
Ilumatobacter_fluminis	13.0	0.02
Muricauda_	13.0	0.02
Isobaculum_melis	13.0	0.02
Coccinimonas_marina	12.0	0.02
Fluviicola_	12.0	0.02

Alteromonas_macleodii	12.0	0.02
Geodermatophilus_	12.0	0.02
Stanieria_cyanosphaera	12.0	0.02
Succiniclasticum_	12.0	0.02
Clostridium_ruminantium	12.0	0.02
Persicobacter_diffluens	12.0	0.02
Bacteroides_	11.0	0.01
Skermanella_	11.0	0.01
Salinisphaera_	11.0	0.01
SC3_	11.0	0.01
Adlercreutzia_	11.0	0.01
Plesiocystis_	10.0	0.01
Candidatus_	10.0	0.01
Bifidobacterium_	10.0	0.01
A17_	9.0	0.01
Rhodopirellula_baltica	9.0	0.01
Rubellimicrobium_	9.0	0.01
Brevibacterium_casei	8.0	0.01
Lactobacillus_hamsteri	8.0	0.01
Acetobacter_aceti	8.0	0.01
Agreia_pratensis	8.0	0.01
Marmoricola_aurantiacus	8.0	0.01

Top Species Classification

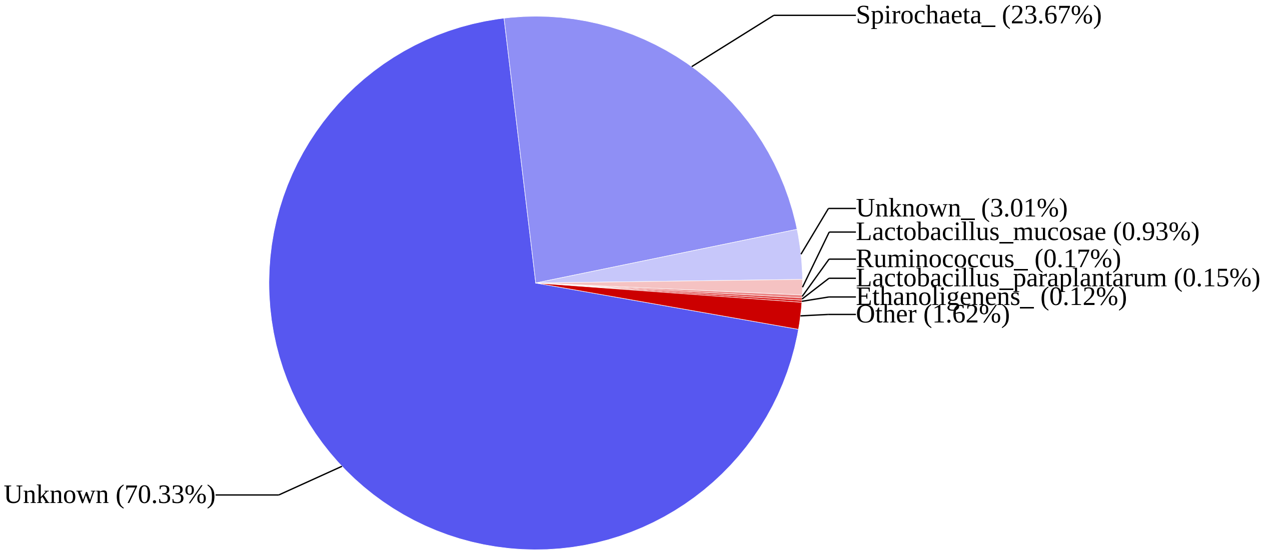

---------- End of report ----------
