# Supplementary material for: Metabolic Profiling, Antiviral Activity and the Microbiome of Some Mauritian Soft Corals
Source: Mar Drugs. 2023 Oct 31;21(11):574. doi: 10.3390/md21110574 (PMC10672535; doi:10.3390/md21110574)
Supplement: Supplementary file 1 [file marinedrugs-21-00574-s001.zip › Supplementary data S5 - L.crassum.pdf]

inqaba biotec™

Africa's Genomics Company

inqaba biotec metagenomic report

Sample Information

Index:	S4
Sample Name:	SF3
Run Name:	230508
Report Date:	Thu May 11 08:41:47 2023

This report contains the summarized metagenomic analysis of 16s/ITS1F gene sequencing. Samples were sequenced on an illumina system (www.illumina.com). Reads were processed through usearch (https://drive5.com/usearch) and taxonomic information was determined based on the Ribosomal Database Project's (http://rdp.cme.msu.edu/index.jsp) 16s database v16 or in the case of ITS1F, the RDP ITS V2 database. Operational Taxonomic Units (OTUs) contributing less than 1% of the total data have been excluded Report generation command used : $create_vsearch_single_sample_pdf_report_illumina.py KT-SF3_S4.merged.filt.otu.table.tsv S4 SF3 230508 16s

Taxanomical Classification

Kingdom Classification

Kingdom	Read Count	%
Bacteria	84301.0	93.79
Unknown	5580.0	6.21

Top Kingdom Classification

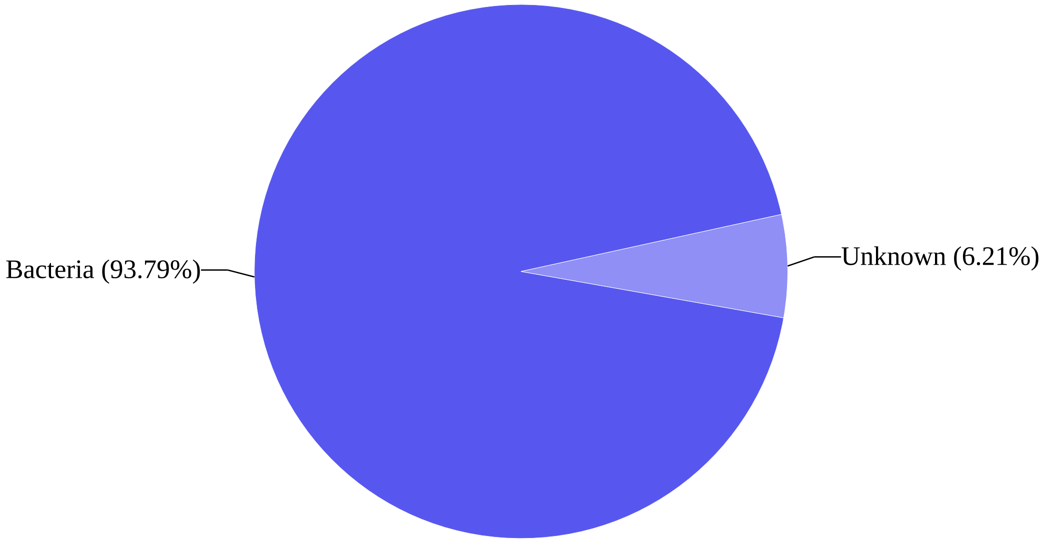

Phylum Classification

Phyla Classification	Read Count	%
Unknown	73986.0	82.32
Firmicutes	9175.0	10.21
Proteobacteria	5082.0	5.65
Bacteroidetes	670.0	0.75
Actinobacteria	395.0	0.44
Verrucomicrobia	143.0	0.16
Spirochaetes	139.0	0.15
Cyanobacteria	94.0	0.10
Planctomycetes	86.0	0.10
Chloroflexi	44.0	0.05
Gemmatimonadetes	35.0	0.04
GN02	14.0	0.02
WS3	13.0	0.01

Top Phylum Classification

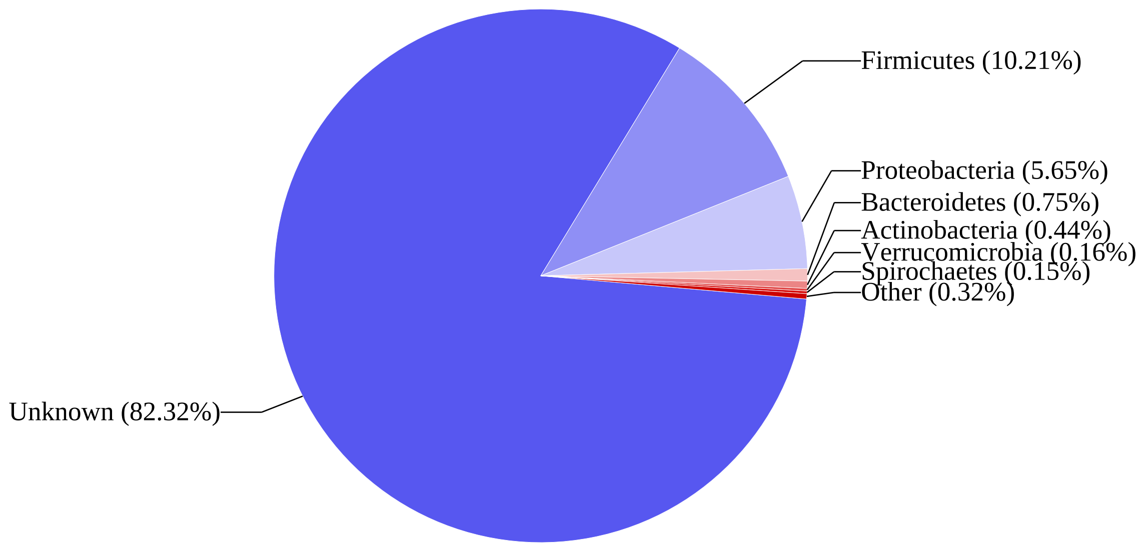

Class Classification

Class	Read Count	%
Unknown	74415.0	82.79
Bacilli	5066.0	5.64
Clostridia	3763.0	4.19
Alphaproteobacteria	3329.0	3.70
Gammaproteobacteria	1359.0	1.51
Flavobacteriia	461.0	0.51
Actinobacteria	395.0	0.44
Betaproteobacteria	359.0	0.40
Spirochaetes	139.0	0.15
Bacteroidia	105.0	0.12
Verrucomicrobiae	102.0	0.11
	94.0	0.10
Oscillatoriophycideae	73.0	0.08
Planctomycetia	55.0	0.06
Gemmatimonadetes	35.0	0.04
Phycisphaerae	31.0	0.03
Deltaproteobacteria	28.0	0.03
Anaerolineae	26.0	0.03
Ellin6529	18.0	0.02
Nostocophycideae	13.0	0.01
PRR	13.0	0.01

Top Class Classification

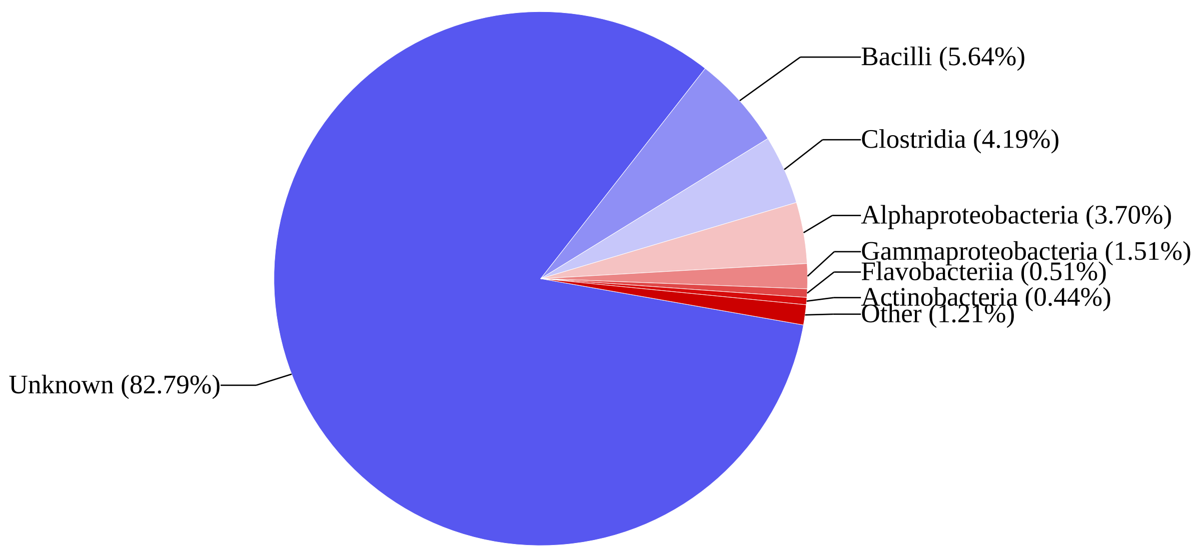

Order Classification

Order	Read Count	%
Unknown	74679.0	83.11
Lactobacillales	4838.0	5.38
Clostridiales	3759.0	4.18
Sphingomonadales	1939.0	2.16
Rhizobiales	1051.0	1.17
Oceanospirillales	550.0	0.61
Flavobacteriales	461.0	0.51
Actinomycetales	395.0	0.44
Pseudomonadales	278.0	0.31
Enterobacteriales	216.0	0.24
Burkholderiales	189.0	0.21
Bacillales	186.0	0.21
Alteromonadales	169.0	0.19
Methylophilales	144.0	0.16
Bacteroidales	105.0	0.12
Verrucomicrobiales	102.0	0.11
Rhodobacterales	94.0	0.10
	76.0	0.08
Caulobacterales	72.0	0.08
Oscillatoriales	71.0	0.08
Rhodospirillales	66.0	0.07
Pirellulales	55.0	0.06
Thiotrichales	48.0	0.05
Spirochaetales	46.0	0.05
Vibrionales	44.0	0.05
Methylacidiphilales	41.0	0.05
Ellin5290	34.0	0.04
Phycisphaerales	31.0	0.03
Myxococcales	28.0	0.03
SBR1031	24.0	0.03
Kiloniellales	16.0	0.02
Nostocales	13.0	0.01
Sediment	13.0	0.01
Gemellales	10.0	0.01
Kordiimonadales	9.0	0.01
Rhodocyclales	9.0	0.01

Top Order Classification

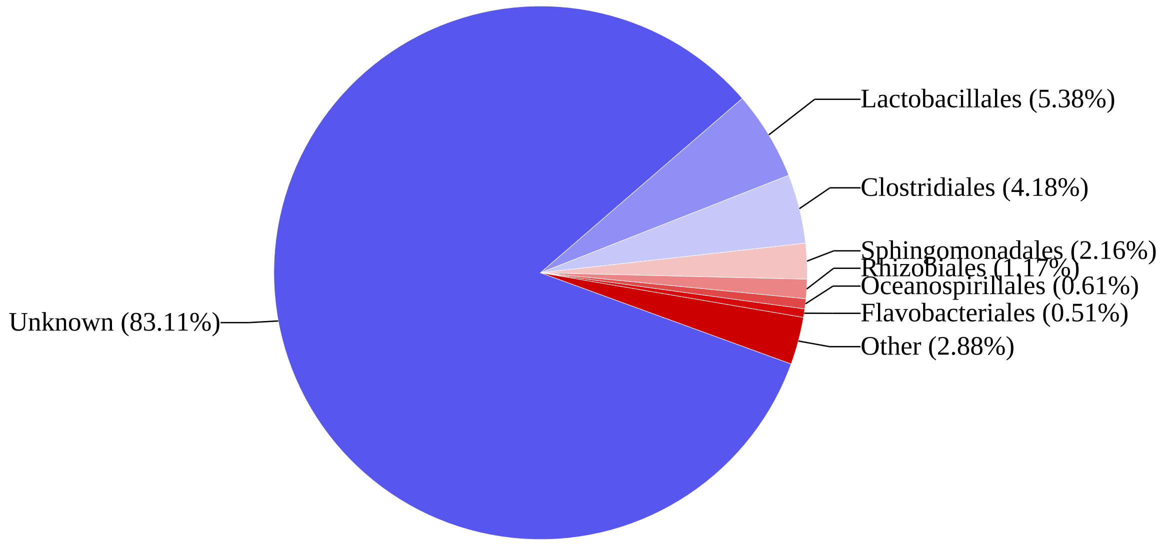

Family Classification

Family	Read Count	%
Unknown	75472.0	84.00
Lactobacillaceae	4636.0	5.16
Lachnospiraceae	3559.0	3.96
Sphingomonadaceae	1885.0	2.10
Methylobacteriaceae	958.0	1.07
Endozoicimonaceae	549.0	0.61
Flavobacteriaceae	308.0	0.34
Enterobacteriaceae	216.0	0.24
Pseudomonadaceae	187.0	0.21
Comamonadaceae	167.0	0.19
Methylophilaceae	144.0	0.16
Alteromonadaceae	137.0	0.15
Propionibacteriaceae	123.0	0.14
Bacillaceae	114.0	0.13
Verrucomicrobiaceae	102.0	0.11
Micrococcaceae	98.0	0.11
Rhodobacteraceae	94.0	0.10
Moraxellaceae	90.0	0.10
Corynebacteriaceae	89.0	0.10
Streptococcaceae	87.0	0.10
Caulobacteraceae	72.0	0.08
Bartonellaceae	69.0	0.08
Pirellulaceae	55.0	0.06
Piscirickettsiaceae	48.0	0.05
Oscillatoriaceae	47.0	0.05
Spirochaetaceae	46.0	0.05
Rhodospirillaceae	43.0	0.05
LD19	41.0	0.05
Aerococcaceae	39.0	0.04
Actinosynnemataceae	32.0	0.04
Phycisphaeraceae	31.0	0.03
Erythrobacteraceae	28.0	0.03
Saprospiraceae	27.0	0.03
A4b	24.0	0.03
Phormidiaceae	24.0	0.03
Cryomorphaceae	24.0	0.03
Clostridiaceae	24.0	0.03
Acetobacteraceae	23.0	0.03
Burkholderiaceae	18.0	0.02

Mycobacteriaceae	18.0	0.02
Planococcaceae	17.0	0.02
Vibrionaceae	16.0	0.02
CV106	13.0	0.01
Bacteroidaceae	12.0	0.01
Chitinophagaceae	10.0	0.01
Brevibacteriaceae	9.0	0.01
Porphyromonadaceae	9.0	0.01
Rhodocyclaceae	9.0	0.01

Top Family Classification

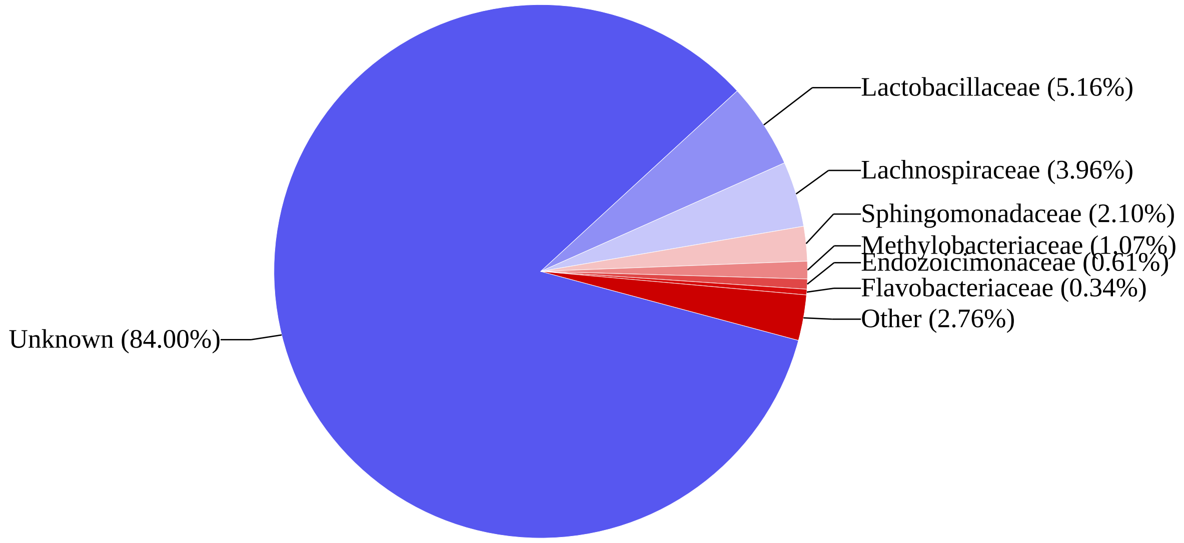

Genus Classification

Genus	Read Count	%
Unknown	80717.0	89.87
Lactobacillus	4605.0	5.13
Sphingomonas	1847.0	2.06
Methylobacterium	942.0	1.05
Endozoicomonas	329.0	0.37
Propionibacterium	122.0	0.14
Corynebacterium	89.0	0.10
Acinetobacter	75.0	0.08
Lyngbya	70.0	0.08
Bartonella	69.0	0.08
Brevundimonas	66.0	0.07
Cloacibacterium	65.0	0.07
Flavobacterium	64.0	0.07
Streptococcus	58.0	0.06
Bacillus	56.0	0.06
Persicirhabdus	51.0	0.06
Rubritalea	48.0	0.05
Kocuria	46.0	0.05
Tenacibaculum	46.0	0.05
Spirochaeta	45.0	0.05
Ascidianibacter	44.0	0.05
Pseudomonas	42.0	0.05
Facklamia	34.0	0.04
Lactococcus	29.0	0.03
Cryomorpha	24.0	0.03
Acetobacter	19.0	0.02
Alteromonas	18.0	0.02
Burkholderia	18.0	0.02
Mycobacterium	18.0	0.02
Candidatus	17.0	0.02
Anaerococcus	16.0	0.02
Enterovibrio	16.0	0.02
Ruegeria	15.0	0.02
BD2	15.0	0.02
Enhydrobacter	13.0	0.01
Anaerospora	13.0	0.01
Bacteroides	12.0	0.01
Novosphingobium	12.0	0.01
Brevibacterium	9.0	0.01
Oceanobacillus	9.0	0.01
Methyloversatilis	9.0	0.01

Top Genus Classification

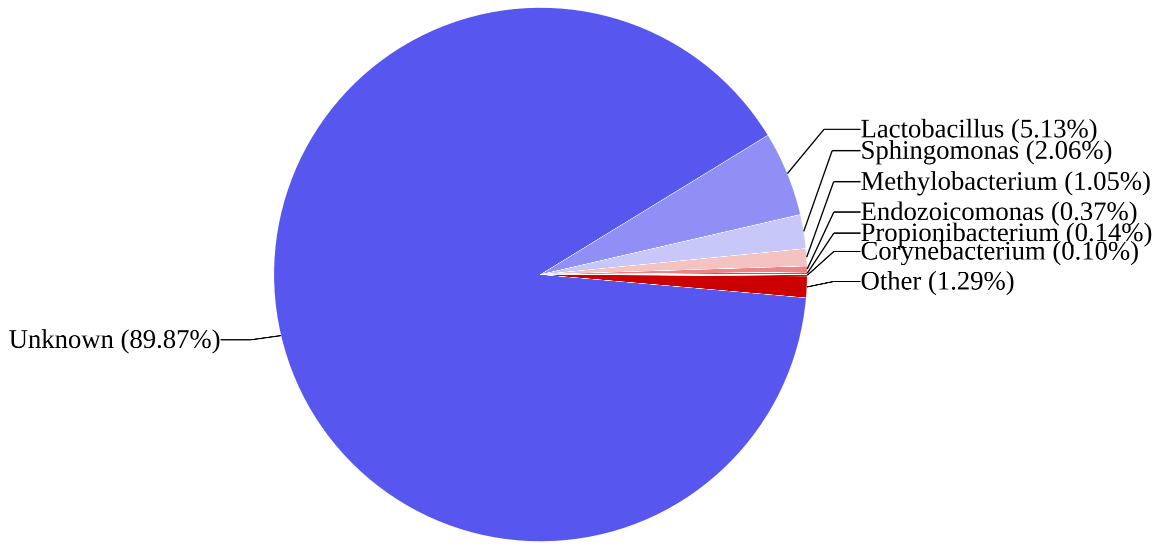

Species Classification

Species	Read Count	%
Unknown	87673.0	97.63
Unknown_	606.0	0.67
Lactobacillus_hamsteri	493.0	0.55
Lactobacillus_mucosae	264.0	0.29
Cloacibacterium_	65.0	0.07
Persicirhabdus_	51.0	0.06
Acinetobacter_guillouiae	49.0	0.05
Rubritalea_	48.0	0.05
Lyngbya_	46.0	0.05
Kocuria_palustris	46.0	0.05
Tenacibaculum_	46.0	0.05
Spirochaeta_	45.0	0.05
Ascidianibacter_aurantiacus	44.0	0.05
Facklamia_	34.0	0.04
Lactococcus_	29.0	0.03
Methylobacterium_hispanicum	23.0	0.03
Cryomorpha_	23.0	0.03
Propionibacterium_acnes	22.0	0.02
Corynebacterium_	22.0	0.02
Acinetobacter_lwoffii	21.0	0.02
Lactobacillus_manihotivorans	19.0	0.02
Alteromonas_macleodii	18.0	0.02
Flavobacterium_	18.0	0.02
Candidatus_	17.0	0.02
Anaerococcus_	16.0	0.02
BD2_	15.0	0.02
Enhydrobacter_aerosaccus	13.0	0.01
Anaerospora_hongkongensis	13.0	0.01
Brevibacterium_	9.0	0.01
Methyloversatilis_	9.0	0.01

Top Species Classification

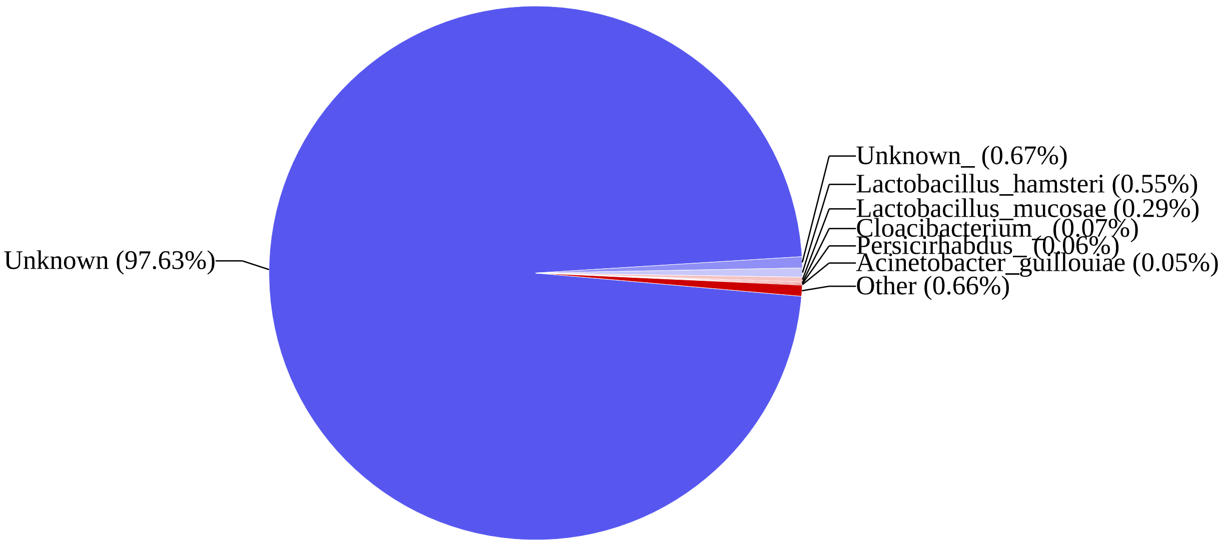

---------- End of report ----------
