# Supplementary material for: Metabolic Profiling, Antiviral Activity and the Microbiome of Some Mauritian Soft Corals
Source: Mar Drugs. 2023 Oct 31;21(11):574. doi: 10.3390/md21110574 (PMC10672535; doi:10.3390/md21110574)
Supplement: Supplementary file 1 [file marinedrugs-21-00574-s001.zip › Supplementary Tables S6-S7.pdf]

Table S6: % yield of each soft corals

Soft corals	% yield			
	Hexane	Ethyl Acetate	Dichloromethane	Methanol
S.polydactyla	8	4	0.8	0.6
C.simplex	3	2.0	0.44	0.3
L.patulum	2.6	1.88	0.36	0.2
L.crassum	10	4	0.42	0.2

Table S7: Crude concentration of each soft corals used

Soft corals	Crude concentration mg/mL			
	Hexane	Ethyl Acetate	Dichloromethane	Methanol
S.polydactyla	38	54.6	28	98
C.simplex	64	16	17.8	157
L.patulum	45.4	11.8	12.8	100
L.crassum	65	58	25.4	74
