# Supplementary material for: Metabolic Profiling, Antiviral Activity and the Microbiome of Some Mauritian Soft Corals
Source: Mar Drugs. 2023 Oct 31;21(11):574. doi: 10.3390/md21110574 (PMC10672535; doi:10.3390/md21110574)
Supplement: Supplementary file 1 [file marinedrugs-21-00574-s001.zip › Supplmentary data S2 - C.simplex.pdf]

inqaba biotec™

Africa's Genomics Company

inqaba biotec metagenomic report

Sample Information

Index:	S5
Sample Name:	FF1
Run Name:	230508
Report Date:	Thu May 11 08:38:03 2023

This report contains the summarized metagenomic analysis of 16s/ITS1F gene sequencing. Samples were sequenced on an illumina system (www.illumina.com). Reads were processed through usearch (https://drive5.com/usearch) and taxonomic information was determined based on the Ribosomal Database Project's (http://rdp.cme.msu.edu/index.jsp) 16s database v16 or in the case of ITS1F, the RDP ITS V2 database. Operational Taxonomic Units (OTUs) contributing less than 1% of the total data have been excluded Report generation command used : $create_vsearch_single_sample_pdf_report_illumina.py KT-FF1_S5.merged.filt.otu.table.tsv S5 FF1 230508 16s

Taxanomical Classification

Kingdom Classification

Kingdom	Read Count	%
Bacteria	100652.0	100.00

Top Kingdom Classification

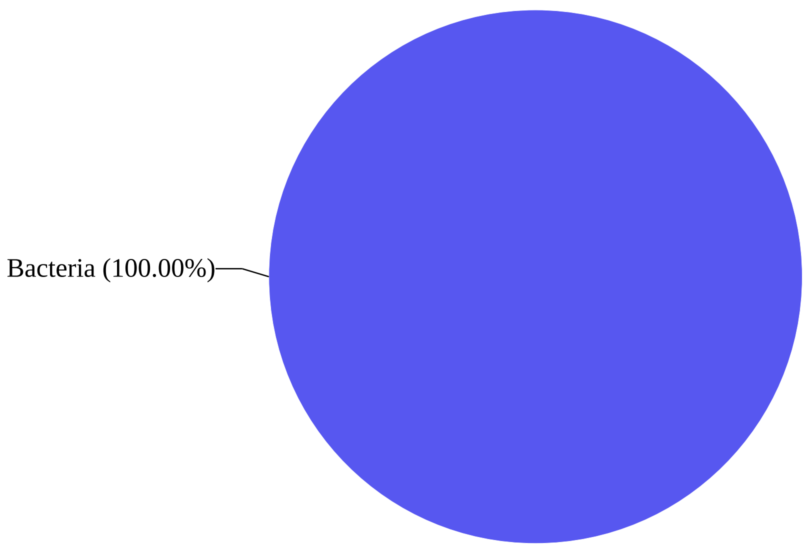

Phylum Classification

Phyla Classification	Read Count	%
Unknown	97150.0	96.52
Proteobacteria	2803.0	2.78
Actinobacteria	370.0	0.37
Firmicutes	127.0	0.13
Bacteroidetes	57.0	0.06
Tenericutes	44.0	0.04
Acidobacteria	42.0	0.04
Planctomycetes	37.0	0.04
Cyanobacteria	27.0	0.03

Top Phylum Classification

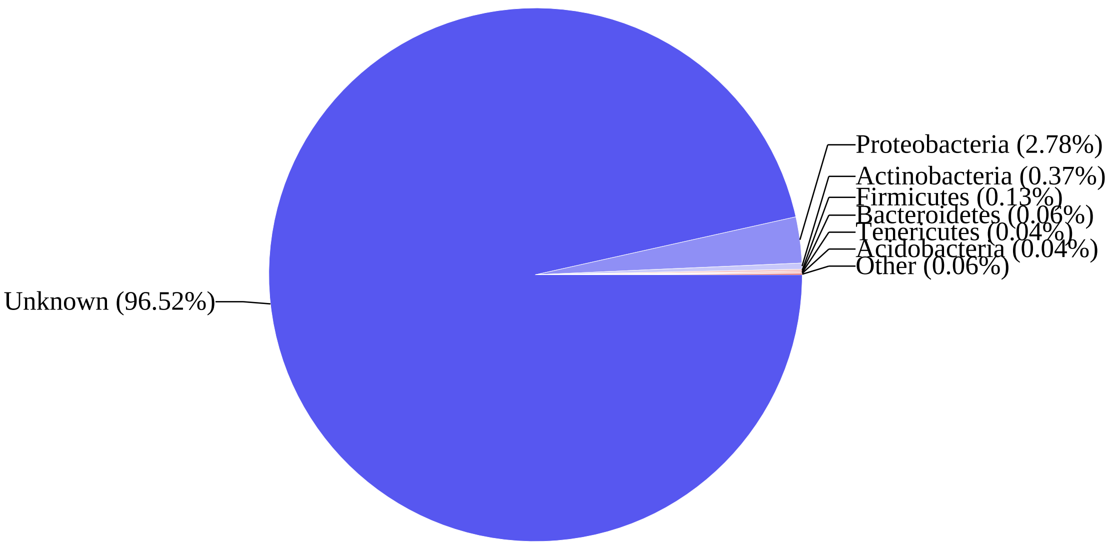

Class Classification

Class	Read Count	%
Unknown	97169.0	96.56
Alphaproteobacteria	2563.0	2.55
Actinobacteria	370.0	0.37
Gammaproteobacteria	176.0	0.17
Bacilli	86.0	0.09
Betaproteobacteria	58.0	0.06
Mollicutes	44.0	0.04
Clostridia	40.0	0.04
Planctomycetia	37.0	0.04
Bacteroidia	28.0	0.03
Acidobacteria	27.0	0.03
Flavobacteriia	25.0	0.02
Sva0725	11.0	0.01

Top Class Classification

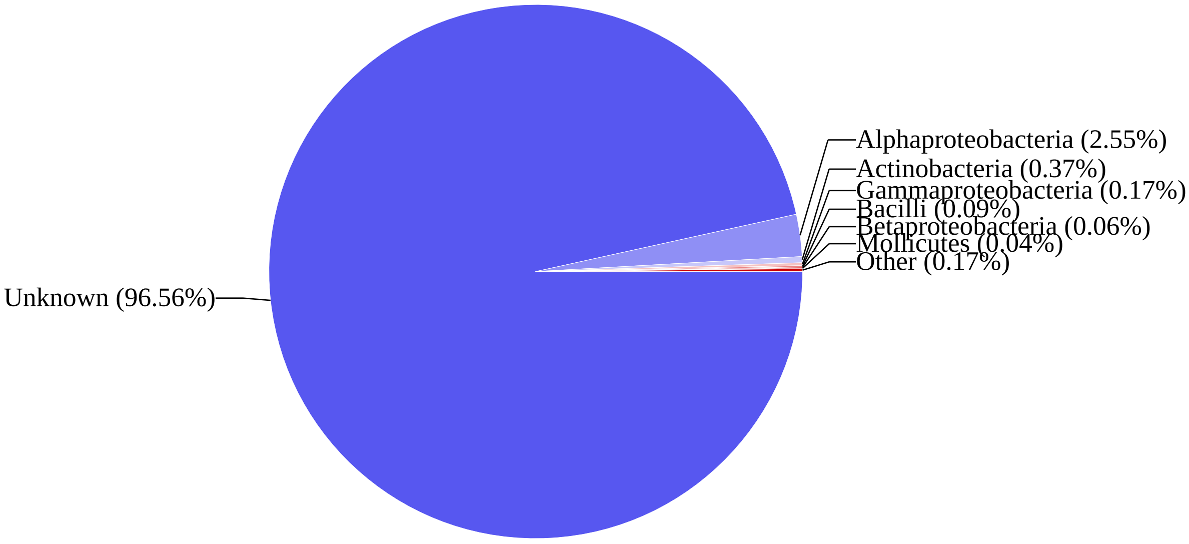

Order Classification

Order	Read Count	%
Unknown	98940.0	98.33
Sphingomonadales	404.0	0.40
Actinomycetales	370.0	0.37
Rhizobiales	322.0	0.32
Pseudomonadales	73.0	0.07
Lactobacillales	65.0	0.06
Oceanospirillales	48.0	0.05
Anaeroplasmatales	44.0	0.04
Burkholderiales	40.0	0.04
	36.0	0.04
Kiloniellales	33.0	0.03
Enterobacteriales	31.0	0.03
Bacteroidales	28.0	0.03
BPC015	27.0	0.03
Flavobacteriales	25.0	0.02
Pirellulales	23.0	0.02
Bacillales	21.0	0.02
Rhodospirillales	19.0	0.02
Legionellales	15.0	0.01
Rhodobacterales	15.0	0.01
Gemmatales	14.0	0.01
Clostridiales	13.0	0.01
Sva0725	11.0	0.01

Top Order Classification

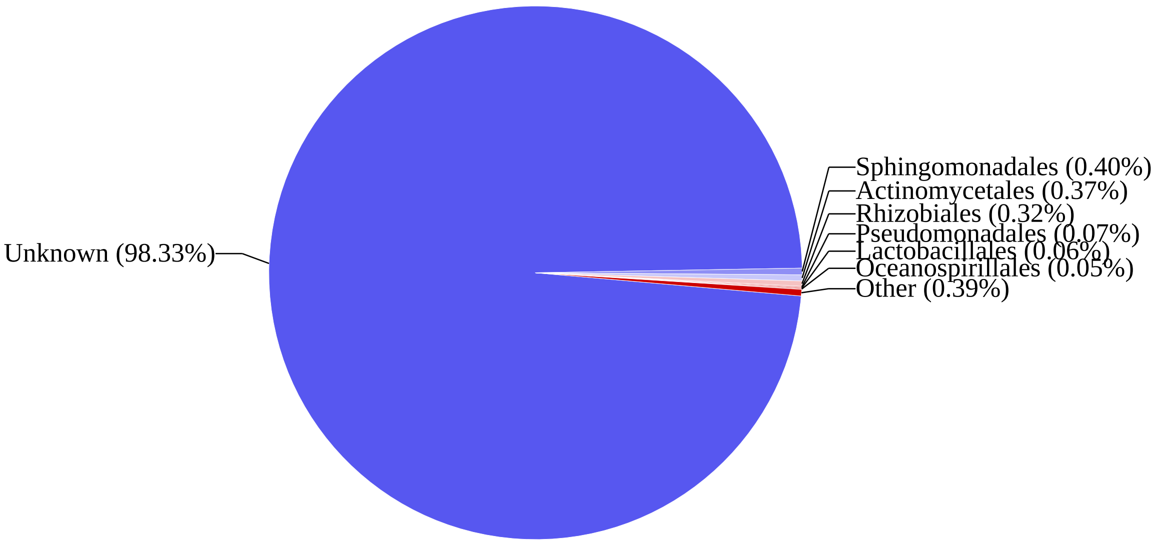

Family Classification

Family	Read Count	%
Unknown	99106.0	98.57
Sphingomonadaceae	401.0	0.40
Methylobacteriaceae	311.0	0.31
Propionibacteriaceae	84.0	0.08
Corynebacteriaceae	76.0	0.08
Pseudomonadaceae	59.0	0.06
Endozoicimonaceae	48.0	0.05
Anaeroplasmataceae	44.0	0.04
Actinomycetaceae	35.0	0.03
Lactobacillaceae	33.0	0.03
Enterobacteriaceae	31.0	0.03
Micrococcaceae	28.0	0.03
Marinilabiaceae	28.0	0.03
Flavobacteriaceae	25.0	0.02
Pirellulaceae	23.0	0.02
Comamonadaceae	21.0	0.02
Intrasporangiaceae	20.0	0.02
Mycobacteriaceae	20.0	0.02
Enterococcaceae	19.0	0.02
Dermabacteraceae	17.0	0.02
Micromonosporaceae	17.0	0.02
Legionellaceae	15.0	0.01
Rhodobacteraceae	15.0	0.01
Microbacteriaceae	14.0	0.01
Isosphaeraceae	14.0	0.01
Moraxellaceae	14.0	0.01
Gordoniaceae	13.0	0.01
Burkholderiaceae	12.0	0.01

Top Family Classification

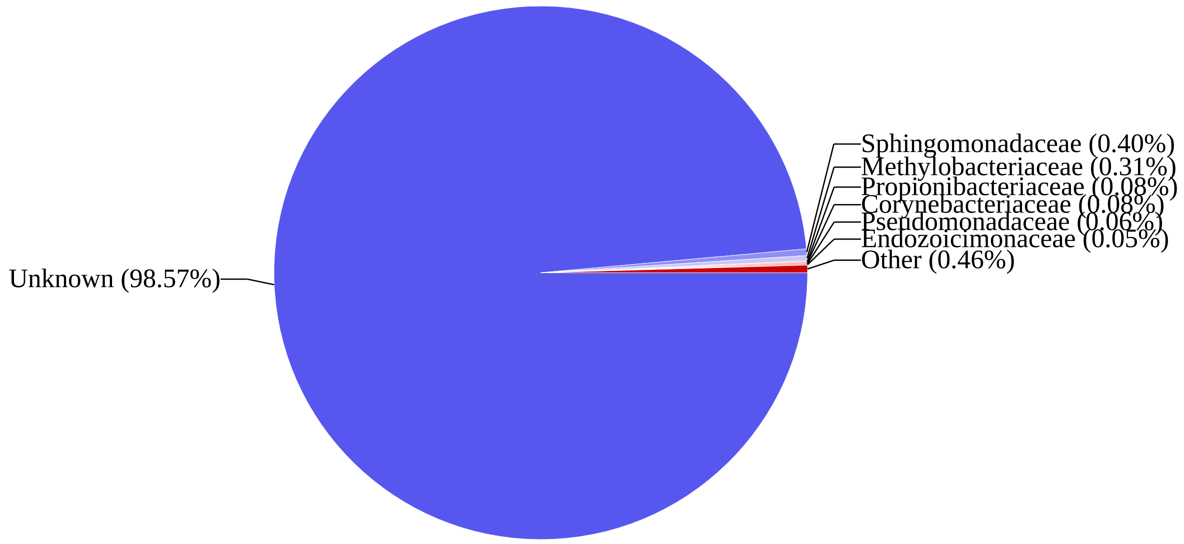

Genus Classification

Genus	Read Count	%
Unknown	99468.0	98.88
Sphingomonas	360.0	0.36
Methylobacterium	307.0	0.31
Propionibacterium	84.0	0.08
Corynebacterium	76.0	0.08
Pseudomonas	35.0	0.03
Actinomyces	34.0	0.03
Lactobacillus	32.0	0.03
Ruminofilibacter	28.0	0.03
Micrococcus	26.0	0.03
Novosphingobium	24.0	0.02
Mycobacterium	20.0	0.02
Enterococcus	19.0	0.02
Legionella	15.0	0.01
Dermabacter	14.0	0.01
planctomycete	13.0	0.01
Gordonia	13.0	0.01
Burkholderia	12.0	0.01
Acinetobacter	11.0	0.01

Top Genus Classification

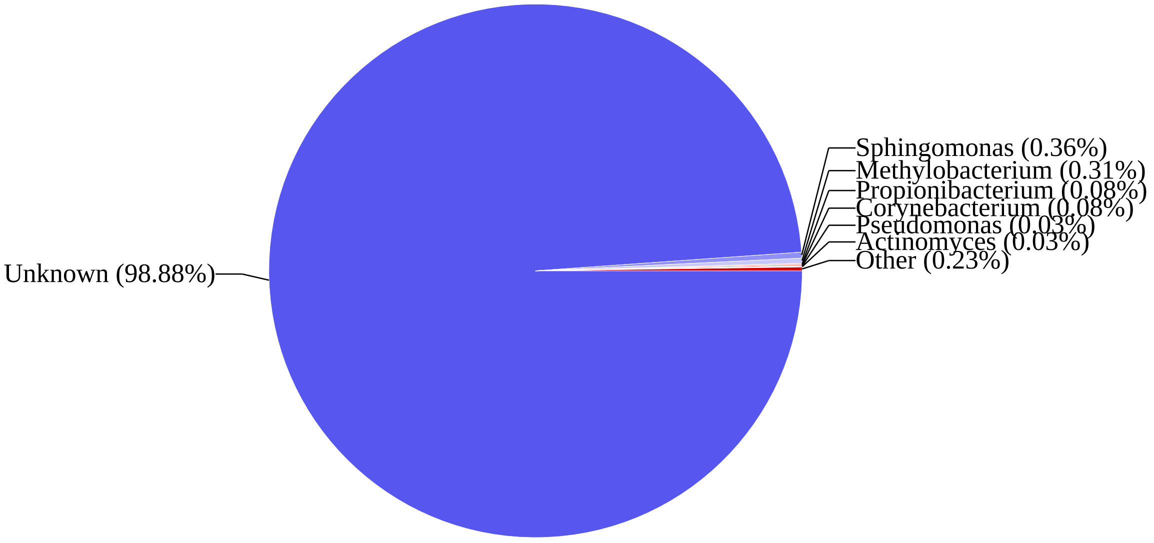

Species Classification

Species	Read Count	%
Unknown	100175.0	99.59
Unknown_	183.0	0.18
Corynebacterium_	63.0	0.06
Actinomyces_	34.0	0.03
Ruminofilibacter_xylanolyticum	28.0	0.03
Micrococcus_	25.0	0.02
Dermabacter_	14.0	0.01
Lactobacillus_mucosae	14.0	0.01
Propionibacterium_acnes	13.0	0.01
Gordonia_	13.0	0.01
Pseudomonas_balearica	12.0	0.01
Mycobacterium_vaccae	11.0	0.01

Top Species Classification

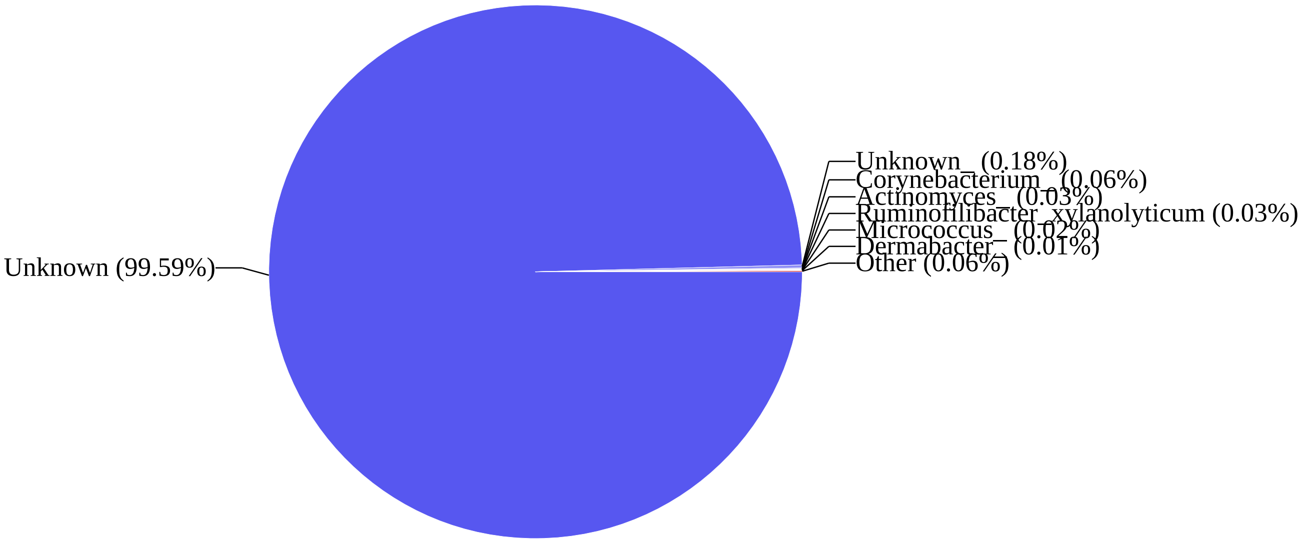

---------- End of report ----------
